# Feasibility and Acceptability of a Dietary Intervention to Reduce Salt Intake and Increase High-Nitrate Vegetable Consumption in Malaysian Middle-Aged and Older Adults with Elevated Blood Pressure: Findings from the DePEC-Nutrition Trial

**DOI:** 10.3390/nu14030430

**Published:** 2022-01-19

**Authors:** Siew Siew Lee, Andrea McGrattan, Yee Chang Soh, Mawada Alawad, Tin Tin Su, Uma Devi Palanisamy, Azizah Mat Hussin, Zaid bin Kassim, Ahmad Nizal bin Mohd Ghazali, Blossom Christa Maree Stephan, Pascale Allotey, Daniel D. Reidpath, Louise Robinson, Devi Mohan, Mario Siervo

**Affiliations:** 1 Global Public Health, Jeffrey Cheah School of Medicine and Health Sciences, Monash University Malaysia, Bandar Sunway 47500, Malaysia; siewsiewlee89@gmail.com (S.S.L.); yee.soh@monash.edu (Y.C.S.); mawada.alawad@monash.edu (M.A.); TinTin.Su@monash.edu (T.T.S.); 2 School of Biomedical, Nutritional and Sports Sciences, Newcastle University, Newcastle upon Tyne NE2 4HH, UK; andrea.mcgrattan@newcastle.ac.uk; 3 South East Asia Community Observatory (SEACO), Jeffrey Cheah School of Medicine and Health Sciences, Monash University Malaysia, Bandar Sunway 45700, Malaysia; daniel.reidpath@icddrb.org; 4 Jeffrey Cheah School of Medicine and Health Sciences, Monash University Malaysia, Bandar Sunway 47500, Malaysia; umadevi.palanisamy@monash.edu; 5 Institute of Medical Science Technology, Universiti Kuala Lumpur, Kajang 43000, Malaysia; azizahmh@unikl.edu.my; 6 District Health Office, Pejabat Kesihatan Daerah (PKD) Segamat, Segamat 85000, Malaysia; drzaid8892@yahoo.com (Z.b.K.); drnizal@yahoo.com (A.N.b.M.G.); 7 Institute of Mental Health, School of Medicine, University of Nottingham, Nottingham NG7 2TU, UK; Blossom.Stephan@nottingham.ac.uk; 8 International Institute for Global Health, United Nations University, Kuala Lumpur 56000, Malaysia; pascale.allotey@unu.edu; 9 International Centre for Diarrhoeal Disease Research, ICDDR, B, Dhaka 1212, Bangladesh; 10 Population Health Science Institute, Newcastle University, Newcastle upon Tyne NE4 5PL, UK; a.l.robinson@newcastle.ac.uk; 11 School of Life Sciences, University of Nottingham Medical School, Nottingham NG7 2UH, UK; Mario.Siervo@nottingham.ac.uk

**Keywords:** dementia, feasibility trial, randomised controlled trial, dietary nitrate, reduced salt

## Abstract

The DePEC-Nutrition trial is a complex dietary and behavioural intervention of salt intake reduction combined with increased high-nitrate vegetable consumption among Malaysian middle-aged and older adults with elevated blood pressure. This study aimed to assess the feasibility and acceptability of the trial. Participants were recruited from the South East Asia Community Observatory (SEACO) database and randomised into one of four groups: (1) low salt; (2) high-nitrate vegetable; (3) combined high-nitrate vegetable and low salt; and (4) control. The intervention included a combination of group counselling sessions, information booklets, reinforcement videos and text messages to modify dietary behaviour. The primary outcomes evaluated were the measures of feasibility and acceptability of (1) recruitment, follow-up attendance and retention; (2) data collection procedures and clinical outcome measures; and (3) individual and combined multi-modal dietary interventions. A total of 74 participants were recruited, and the 10-month retention rate was 73%. Data collection procedures were acceptable with minimal missing data. All intervention strategies were feasible and acceptable, with group counselling being the most acceptable strategy. This study provides important insights into improving the screening process of participants, facilitating their access to the research facilities and refining the measurement protocols and dietary recommendations, which are instrumental in formulating the design of a full-scale definitive DePEC-Nutrition trial.

## 1. Introduction

Dementia is a neurodegenerative disease characterised by a progressive and significant impairment of cognitive and physical function [1]. The pathogenesis of dementia is complex and linked to the multi-factorial contribution of metabolic and vascular changes, genetics and environmental factors, which occur alongside age-related changes in brain structure and function [2,3]. Globally, approximately 50 million people have dementia and this number is projected to triple by 2050, with 70% of dementia cases occurring in low- and middle-income countries (LMICs) [4].

A certain degree of overlap characterises the risk factor profiles of cardiovascular diseases (CVD) and dementia, and factors such as age, smoking, obesity, physical inactivity and depression are significant contributors to both conditions [5]. High blood pressure is an important modifiable risk factor for both CVD and dementia [6,7,8,9], and evidence from clinical trials has reported significant associations between decreased blood pressure, improved cognition, and reduced CVD [10,11] and dementia risk [12,13,14]. At the same time, interventions focused on dietary changes have been shown to have lowering effects on blood pressure and are also linked to a reduced risk of dementia. The Mediterranean diet and the Dietary Approach to Stop Hypertension (DASH) are examples of recognised healthy dietary patterns with strong evidence-based protective effects against both cardiovascular and dementia risk [15,16,17]. Sodium and inorganic nitrate (via the formation of nitric oxide (NO)) are two of the key protective nutrients with health-promoting properties via mechanisms involving the control of vascular tone, fluid balance and neurotransmission [18,19].

Observational studies have reported an association between a high inorganic nitrate intake with a reduced risk of coronary heart disease and cardiovascular mortality [20,21,22], and the effect was more prominent in younger age groups. In addition, 24-h urinary inorganic nitrate concentrations, measured as an objective marker of dietary nitrate intake, were indirectly associated with resting systolic blood pressure among older Italian subjects recruited as part of the InChianti cohort [23]. Several clinical trials have also reported the blood pressure lowering effects of dietary nitrate supplementation [24,25,26,27,28,29,30], and the effect size of interventions on systolic and diastolic blood pressure have been summarised in a recent meta-analysis [31]. Research has demonstrated that consuming foods rich in inorganic nitrate increases NO production [29,32] and has positive effects on brain function [33]. In particular, improvements in cognitive function (executive function) and motor skills following dietary nitrate supplementation have been observed, which appears to be mediated by augmented cerebral blood flow and the efficiency of neuronal cellular metabolism [34,35]. The modification of dietary nitrate intake is, therefore, a potential target for reducing dementia risk in a high-risk population with elevated blood pressure.

Observational studies have consistently shown significant associations between high sodium intake and increased blood pressure [36,37], which have been confirmed in several clinical trials reporting the lowering effects of low salt diets on blood pressure [38,39]. A recent meta-analysis reported a significant association between blood pressure reduction and a lower risk of cognitive impairment and incident dementia [40]. However, a small number of epidemiological studies have observed mixed associations between sodium intake and cognitive performance in older adults [41,42,43,44,45].

There are currently limited data on effective multi-modal dietary and lifestyle interventions that prevent cognitive decline and dementia in LMICs [46]. The DePEC (Dementia Prevention and Enhanced Care)-Nutrition trial is a complex dietary and behavioural intervention evaluating the feasibility of the combination of salt intake reduction and increased high-nitrate vegetable consumption among middle-aged and older Malaysian adults with elevated blood pressure [47]. This trial was designed according to the Medical Research Council (MRC) guidelines for developing and evaluating complex interventions. The guidelines highlight the importance of practical and methodological difficulties in such interventions, which need to be identified through feasibility testing before evaluating the efficacy of full-scale definitive interventions [48]. The present study aimed to assess (1) the feasibility of screening, recruitment, follow-up and retention rate, (2) the suitability and acceptability of data collection procedures and clinical outcome measures within the DePEC-Nutrition trial, and (3) the feasibility and acceptability of long-term (>6 months) individual and combined multi-modal dietary interventions aimed at modifying dietary nitrate (increase) and sodium (decrease) intake in middle-aged and older Malaysian adults with elevated blood pressure.

## 2. Materials and Methods

### 2.1. Study Design

The DePEC-Nutrition trial was a randomised, controlled 2 × 2 factorial nutritional intervention conducted between June 2019 and December 2020. This study was approved by the Monash University Human Research Committee (Project ID: 17864) and the Malaysian Medical Research Ethics Committee (NMRR-19-617-45916), and was registered with the clinical trial registry (ISRCTN47562685). Detailed information on the study design and visits can be found elsewhere [47].

### 2.2. Participants and Recruitment

Potential participants were identified from the South East Asia Community Observatory (SEACO) database: a health and demographic surveillance site (HDSS) within a middle-income community in Segamat, Johor, Malaysia. The following eligibility criteria were applied: (1) aged 50–75 years; (2) resided within a 5 km radius of the study site, Health Clinic Sungai Segamat; (3) systolic blood pressure of 120–159 mmHg or diastolic blood pressure 80–99 mmHg or diagnosed hypertensive; and (4) Body Mass Index (BMI) > 18.5 kg/m^2^. A list of participants who matched these eligibility criteria was generated from the database. Participants were randomly selected and approached via a telephone call or home visit to arrange a screening visit to confirm their eligibility. If they agreed to participate in the study, a home screening visit was arranged. The detailed study inclusion and exclusion criteria are listed in Table A1 in Appendix A.

During the screening visit, participants first signed an informed consent form for participation. Information on demographics, comorbidities, medication use and history, cognitive function (Mini-Mental State Examination, MMSE) [49], body weight, height and resting blood pressure were collected. Eligible participants were then invited to take part in the study, and a baseline assessment visit was arranged. If participants declined to take part in the study, they were asked for a specific reason for not participating.

### 2.3. Dietary Interventions

Participants were randomised into one of four groups in a factorial design: (1) low salt consumption; (2) high-nitrate vegetable consumption; (3) combined high-nitrate vegetable consumption plus low salt consumption; or (4) a control group. Participants allocated to the intervention groups received the DePEC-Nutrition intervention, which consisted of the five strategies outlined below. All intervention strategies were delivered in the participants’ preferred language (English/Malay/Chinese), except for group nutritional counselling, which was offered in Malay. A copy of the intervention resources is available in the Online Supplementary Material.

1. **Group nutritional counselling** was delivered during the baseline clinic visit by a trained medical officer using PowerPoint slides and practical activities. Each session lasted between 1–1.5 hours. The topics addressed at the counselling session included health benefits, sources, recommended intakes and practical tips related to the allocated intervention of increasing dietary nitrate and/or reducing salt intake. Participants in the low salt group were advised to consume less than 5 g of salt in accordance with the recommendations from the World Health Organization (WHO) [50]. Advice was delivered on how to reduce salt intake during home cooking, eating out and shopping for food (reading food labels). Participants in the high-nitrate groups were recommended to consume vegetables rich in dietary nitrate at least three times per week or more to achieve an intake of approximately 1000–1500 mg of dietary nitrate per week. A list of commonly consumed nitrate-rich vegetables (i.e., broccoli, cabbage, spinach, cauliflower, lettuce and eggplant) was provided in the information booklet, along with the recommended portion size. In addition, practical tips for increasing dietary nitrate intake and retaining the nitrate content in the vegetables when cooking were also provided.
2. **Information booklets** were provided during the group counselling sessions. In addition to the detailed information discussed during the sessions, the booklet contained recipes of low salt and/or high-nitrate meals that participants could prepare at home. The booklet also provided several sources and links for further information.
3. **A salt measuring spoon** (Atila GmbH, Neidenstein, Germany, see the picture in the Online Supplementary Material) was provided to the participants who were randomised into the salt interventions (low salt and combined high-nitrate vegetable plus low salt consumption groups) to support them in understanding portion sizes and measuring salt intake. The measuring spoon had a dual side with nine adjustable scales (0.5, 1, 2, 3.5, 5, 7, 9, 11 and 13 g). Participants were taught how to use the spoon during the group counselling sessions.
4. **Biweekly text messages** were sent to all participants in the intervention groups. The text messages included educational messages and reminders of the key dietary behaviour changes to encourage adherence.
5. **Reinforcement video messages** were delivered to the participants in the intervention groups at the interim visits to remind participants of the key dietary advice that was discussed during the baseline counselling sessions. The reinforcement videos were hosted on the YouTube platform and only viewable to those who had access to the video link. Video clip links were sent to the participants via WhatsApp (WhatsApp Inc., Mountain View, CA, USA).

Participants in the control group were provided with an information booklet during their baseline clinic visit. The booklet contained the Malaysian Food Pyramid and 11 key messages on healthy eating and lifestyle recommendations derived from Malaysian dietary guidelines [51]. Participants were reinforced with similar key messages via the WhatsApp application during the interim 3 visit (8 months) of the study.

### 2.4. Randomisation

The randomisation of individual participants into a particular trial group was undertaken once they passed the screening visit and confirmed their participation in the trial. A block randomisation method with a block size of four was performed. A block randomisation list was generated using R software (randomizeR) [52] by a member of the research team who was not involved in data collection.

### 2.5. Study Procedures

Baseline assessments were conducted in person over two visits (first at the participant’s home and then at the health clinic) and within two days. During the home visit, the data collectors explained the study procedures and obtained written informed consent for participation in the trial. Participants were asked about any newly diagnosed diseases and prescriptions or changes in their medication following their last visit during screening to further confirm their eligibility. Eligible participants were then provided with instructions and containers to collect 24-h urine samples and return them when attending the clinic visit. Participants attended the clinic visit in the morning between 8 a.m. and 10 a.m., and they were asked to refrain from strenuous exercise and from consuming stimulating substances (i.e., coffee, tea). A number of assessments were conducted, including resting blood pressure, body composition, 24-h dietary recall, Food Frequency Questionnaire (FFQ) and the collection of biological samples (blood, dried blood spot, saliva, salivary strips and spot urine samples). Detailed information on these assessment methods has been published elsewhere [47].

At interim 1 (2 months), reinforcement messages were provided to participants in the intervention groups to enhance compliance with the intervention. Adherence questionnaires were administered at the same time. At the interim 2 visit (4 months), another set of reinforcement messages was sent to the intervention groups, while adherence questionnaires were administered to all participants, including those in the control group. In addition, at the interim 3 visits (8 months), all participants were provided with videos messages to reinforce the key dietary messages for their respective interventions. A feedback questionnaire was administered at the end of the study to obtain detailed feedback on the overall study process.

### 2.6. Outcomes

The primary outcomes were to evaluate the feasibility of the recruitment, follow-up, retention, data collection procedures, clinical outcome measures and interventions to inform the design and delivery of a full-scale DePEC-Nutrition trial. Secondary outcomes included clinical and nutritional measures: blood pressure; Montreal Cognitive Assessment (MoCA) test; auditory verbal learning test (AVLT); 24-h recall; Food Frequency Questionnaire (FFQ); and nitrate concentrations in urine, saliva and blood samples. The Bahasa Malay version of the MoCA (intra-class correlation coefficient = 0.81) [53] and AVLT (factor loading 0.66 to 0.98) [54] have been previously validated among the Malaysian population. In addition, the FFQ is widely used in the Malaysian Adult Nutrition Survey (MANS) 2014 [55]. These are key for informing the calculation of sample size and recruitment strategies in the definitive DePEC-Nutrition RCT. A mixed-method approach was used, including quantitative measures of feasibility, acceptability and clinical outcomes and a qualitative evaluation of the experience of the study participants and data collectors. A detailed description of the outcome measurements is provided in Table A2 in Appendix A. The primary outcomes were measured at three time points: interim 1 (2 months); interim 2 (4 months); and end of study (6 month). Self-rated acceptability data collection procedures and the usefulness of the interventions strategies and materials were assessed using a questionnaire developed and pre-tested by the research team. The questionnaire included a mixture of open-ended and close-ended questions. A copy of the questionnaire is available in the Online Supplementary Material.

### 2.7. Protocol Changes Implemented during the COVID-19 Outbreak

The baseline assessment started in November 2019. In March, when the coronavirus disease 2019 (COVID-19) was declared as a global pandemic by the World Health Organization, this trial was in the baseline assessment and interim 1 stage (for some of the participants). By 13 March 2020, two days before the Malaysian government announced a movement restriction order, a total of 97 participants had consented to participate, 74 participants had completed the baseline assessment, and 18 participants had completed the interim 1 assessment. To allow the continuity of this trial during COVID-19, the original DePEC-Nutrition trial’s protocol was adapted, as follows:

1. Methodology of data collection
  The data collection at interim 1, interim 2 and the end of study visits was initially planned to be conducted in person at the participant’s home. However, due to the COVID-19 outbreak, the need for social distancing and the movement restriction imposed by the Malaysian government, data collection by home visit was not possible. Hence, to continue the study during the COVID-19 pandemic, the data collection at interim 1, 2 and the end of study visits were conducted via telephone interview. The collection of information related to the primary outcomes of evaluating the acceptability and feasibility of the intervention was prioritised. The measurement of the secondary outcomes (i.e., physical assessment and biological samples) was not performed.
2. Timeframe
  Initially, the planned duration of the DePEC-Nutrition feasibility study was six months, with interim 1 in the second month and interim 2 in the fourth month after the baseline. The shift from face-to-face data collection to telephone-based data collection required an extension of the study in order to implement the necessary changes to the protocol, including (1) obtaining approval from the ethics committee, SEACO and Monash University Malaysia on the amendment of the study protocol, (2) the development of the telephone-based data collection protocol, including the adaptation of the questionnaires, and (3) the re-training of the data collectors. Therefore, the total duration of the study was extended to 10 months and an additional interim 3 was added at month eight.
3. Intervention delivery
  The reinforcement was originally planned to be delivered by the data collectors during the interim 1 and 2 visits. Due to the COVID-19 outbreak and the need for social distancing, video reinforcements were instead sent to each participant through WhatsApp. Participants without a mobile phone number were informed of the video content by the data collectors or had their close relative receive the video message on their behalf. In addition, the videos were also copied onto a compact disc and posted to participants who did not have access to a mobile phone. The reinforcement video included a comprehensive review of the key dietary messages delivered through nutritional counselling at the start of the study and provided specific information on adapting and making dietary changes during the pandemic.

### 2.8. Sample Size

The initial recruitment target was 30 participants per group. With a dropout rate of ≤20%, it was expected that more than 100 participants would complete the trial. This pragmatic sample size was based on guidelines by Whitehead et al. (2016) [56], which indicate that a sample size of greater than 25 individuals per group would provide a 90% power to detect a small effect size ranging between 0.1 and 0.3 for changes in blood pressure.

### 2.9. Data Presentation and Statistical Analysis

Continuous data were checked for normality using the Shapiro–Wilk test and skewness z-score [57]. The mean and standard deviation (SD) were presented for normally distributed variables, whereas the median and interquartile range (IQR) were presented for skewed variables. Categorical variables were reported as frequency and proportions. One-way ANOVA or Kruskal–Wallis tests were used to compare baseline characteristics between intervention groups. Categorical variables were analysed by Chi-squared or Fisher’s exact tests. Screening, eligibility, consent, retention, completion and missing data rates were described as numbers and proportions. Reasons for non-participation, non-responses and the acceptability of study visits were described in a narrative format. The acceptability of the reduced salt and high-nitrate vegetable consumption interventions was tested by contrasting two a priori factors based on the 2 × 2 factorial design: (1) a low salt factor (low salt consumption and combined high-nitrate vegetable plus low salt consumption) vs. no low salt (high-nitrate vegetable consumption and control groups) and (2) a high nitrate factor (high-nitrate vegetable and combined high-nitrate vegetable consumption plus low salt groups) vs. non-nitrate (low salt and control groups). All statistical analyses were performed using SPSS version 26.0 (SPSS Inc., Chicago, IL, USA). Statistical significance was set at *p* < 0.05.

## 3. Results

### 3.1. Recruitment, Follow-Up Response and Retention

#### 3.1.1. Recruitment

**Figure 1:**
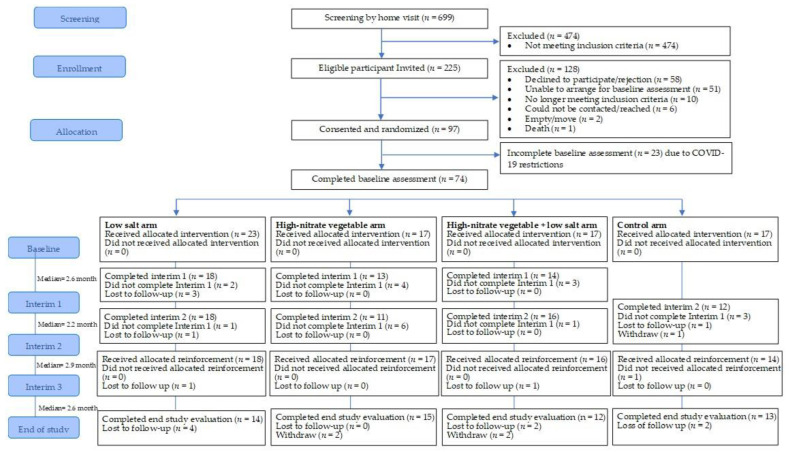
The flow of participants through the pilot study.

Figure 1 shows the flow of the participants through the trial. Of the 699 potential participants who were screened, 225 (32.2%) participants were considered eligible. The major reasons for ineligibility were: medication use (31.6%, 150/474); acute or chronic medical condition (15.6%, 74/474); and insulin therapy (10.8%, 51/474) (Supplementary Material, Table S1). Of the 225 eligible participants, 97 (43%, 97/225) consented to participate in the study, almost a quarter (51/225, 23%) were unable to arrange the baseline assessment and about a quarter (26%, 58/225) of participants did not agree to participate. The major reasons for not agreeing to participate included: (1) lack of interest in the study (62%, 36/58); (2) not a suitable time (14%, 8/58); and (3) the study visits were too frequent (12%, 7/58) (Supplementary Material, Table S2). The proportion of participants agreeing to participate was significantly lower for Malay participants compared to Chinese participants (57.3% vs. 78.4%, *p* = 0.031), single vs. married (45.7% vs. 67.5%, *p* = 0.019) and alcohol consumers compared to non-alcohol consumers (100% vs. 60%, *p* = 0.014) (Supplementary Material, Table S3).

The baseline assessments and recruitment were stopped due to restrictions imposed by COVID-19, and therefore, reaching the target sample size (*n* = 120) was not possible. Of the consenting participants, 74 were recruited and completed the baseline assessment within the restricted time frame (26 November 2019 to 13 March 2020). Additional logistic challenges related to scheduling baseline visits, included (1) difficulties in contacting potential participants, (2) the burden of research phone calls, and (3) work commitments or child-minding duties.

#### 3.1.2. Follow-Up Response Rate and Retention

The overall follow-up attendance rate for interim 1 and interim 2 were 79% (45/57) and 77% (57/74), respectively. There were no related or unexpected adverse events reported. The median intervention period was 10.5 months (Range = 9.4–11.7 months). At the end of the study, 54 of the 74 participants completed the end study evaluation and the overall retention rate was 73%. There was no evidence that the retention rate differed between the study groups (low salt: 61% (14/23); high-nitrate vegetable: 88% (15/17); combined high-nitrate vegetable plus low salt: 71% (12/17); and control: 76% (13/17); Fisher’s exact *p*-value = 0.291). A total of 20 participants (27%) dropped out of the study and reasons included being uncontactable (*n* = 10), no longer being interested in the study (*n* = 5), work or childcare commitments (*n* = 4) and moving out of the study location (*n* = 1). Participants retained until the end of the study had higher MMSE (*p* = 0.022) and MoCA scores (*p* = 0.023) than participants who dropped out (Supplementary Material, Table S3).

#### 3.1.3. Characteristics of Study Participants 

A total of 74 participants were enrolled in the DePEC-Nutrition study and randomised into one of the four intervention groups: low salt group (*n* = 23); high-nitrate vegetable group (*n* = 17); high-nitrate vegetable and low salt group (*n* = 17); and control group (*n* = 17). The baseline characteristics of the study participants are presented in Table 1. The mean ± SD age of participants was 61.6 ± 6.7 years. About half of the participants worked full-time (35.1%) or part-time (12.2%). Almost half of the participants (48.6%) had secondary education, and the mean MoCA score was 20.8 ± 4.0. All characteristics were similar between the intervention and control groups. Based on 24-h dietary recall data, the baseline median total energy and sodium intake was 1797 (Q_1_–Q_3_: 1229–2477) kcal/day and 2777 (Q_1_–Q_3_: 1813–4330) mg/day, respectively. An analysis of the FFQ baseline data showed that the median frequency of intake for green leafy vegetables was 3.0 (Q_1_–Q_3_: 1.0–7.0) times per week, with 37.5% (27/72) of the participants consuming green leafy vegetables daily.

### 3.2. Suitability and Acceptability of Data Collection Procedures and Outcomes Measures

#### 3.2.1. Study Visits

Data collectors considered home visits appropriate and safe. Similarly, the majority of participants reported that the number (90.7%, 49/54) and duration of the visits (84.9%, 45/53) were appropriate and convenient (96.2%, 50/52) (Table 2). Six participants (6/53, 11.3%) mentioned that the duration of the clinic visits was long for them and their dependents. Similarly, five of the data collectors mentioned that the duration of the home and clinic visits ranged from 30 to 120 min and in some instances, this was too long. In addition, five of the data collectors felt that clinics were not a suitable setting for data collection because the clinic was crowded and the process may have been uncomfortable for the participants. In addition, assessments were performed in different rooms within the clinic and participants were required to frequently move to the different stations.

#### 3.2.2. Questionnaire

At baseline, most clinical outcome measures were completed by the participants without significant difficulty apart from the Trail Making B test, which was not completed by 10 illiterate participants who could not recognise alphabets (*n* = 6) or had difficulties understanding the instructions (*n* = 4). Data collectors also indicated that the duration of baseline assessments may have been too long and that this may have been stressful for some participants (Supplementary Material, Table S4).

Overall, 43 (58%, 43/74) participants provided a complete set of follow-up and end of study data, and 65 (88%, 65/74) provided at least either follow-up (interim) data or end of study data. Participants answered the majority of the questions with very little missing data (<5%). However, data collectors indicated that it was challenging to explain the questions to participants over the phone.

“*Phone interviews were quite challenging when the respondent can’t imagine or even understand the score and questions given as the choice of the answer was confusing*”.

(Data collector 3)

#### 3.2.3. Biological Sample Collection and Physical Measurements

**Figure 2:**
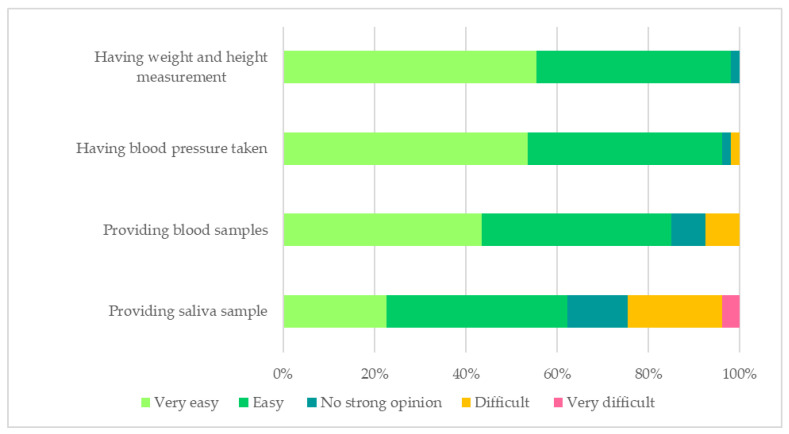
The acceptability of the baseline assessments. Missing information: providing blood samples (*n* = 1); providing saliva sample (*n* = 1).

During the baseline assessment, most of the participants (96%, 71/74) provided blood sample, dried blood spot sample, saliva sample, 24-h urine, spot urine samples, blood pressure and anthropometry data. Specifically, 61 participants provided complete dried blood spots while 64 collected complete matched 24-h and spot urine samples according to the predefined criteria. Overall, the anthropometry and blood pressure measurement and blood collection procedures were acceptable for most of the participants (Figure 2). Similarly, the data collectors found that the methods for collecting blood pressure, anthropometry data and biological samples (blood samples, dried blood spot sample, 24-h urine, spot urine) were easy. About 30% of participants indicated that providing the saliva sample was difficult, which was also reflected in the feedback provided by the data collectors.

### 3.3. Feasibility and Acceptability of Various Nutrition Education Strategies

All participants allocated to the three interventions groups (100%, 47/47) attended the group counselling sessions (Table 3). More participants read the text messages (80%, 33/41) and watched the video (85%, 35/41) than read the information booklet (69%, 31/45). Participants who did not have access to messaging services received the reinforcement via a telephone call (Interim 1: *n* = 4; Interim 2: *n* = 11; and Interim 3: *n* = 7) or a compact disc with the video was sent via post (Interim 3: *n* = 2). Half of the participants from the intervention groups (51%, 21/41) claimed that they tried the recipes in the booklet provided. About one third (37%, 15/41) claimed that they accessed additional material suggested in the further reading section.

**Figure 3:**
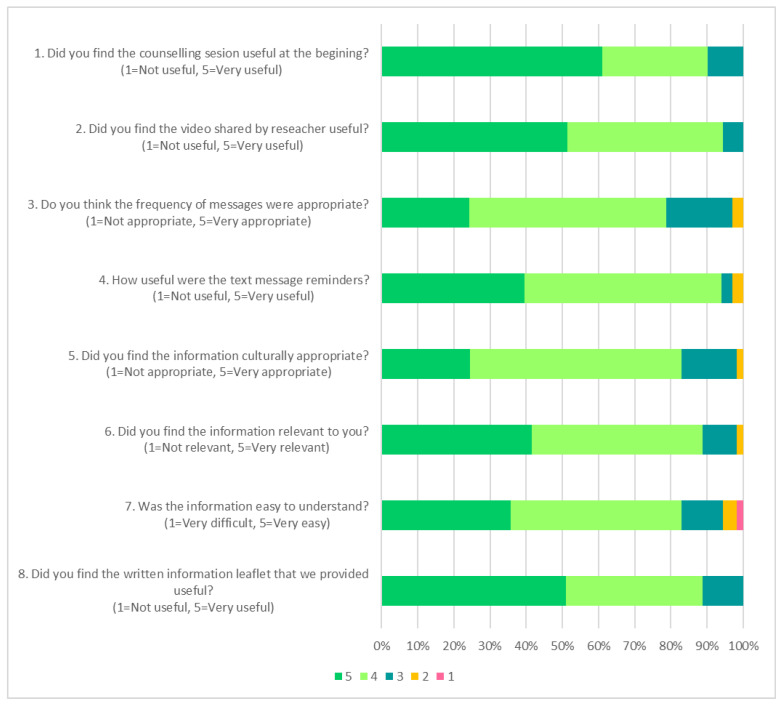
The acceptability and suitability of the intervention materials. The response rate for each question was: 1 (*n* = 41/41); 2 (35/41); 3 (33/41); 4 (33/41); 5 to 8 (53/54). Question 1 to 4 was only applicable to the intervention groups (*n* = 41) and responded to by those engaged with the intervention strategies.

The majority of participants indicated the usefulness of the group counselling (90%, 37/41), text messages (94%, 31/33), videos (94%, 33/35) and the information booklet (89%, 47/53) (Figure 3). While most participants agreed that the information booklet was easy to understand (83%, 44/53), relevant (89%, 47/53) and culturally appropriate (83%, 44/53), a small proportion thought that the information in the booklet was moderately useful (11%, 6/53), difficult or very difficult to understand (6%, 3/53), irrelevant (2%, 1/53) and culturally inappropriate (2%, 1/53). Those who rated the information in the booklet as moderately useful and irrelevant were from the high-nitrate vegetable (*n* = 5) or high-nitrate vegetable plus low salt groups (*n* = 1). This is consistent with the qualitative data, in which two Chinese participants from the high-nitrate vegetable group mentioned that the booklet contained mostly recipes tailored for the Malay community. 

Overall, a significant number of participants mentioned the usefulness of the group counselling sessions, text messages and the information booklet for providing information and knowledge and serving as a reminder to them to comply with the relevant intervention. Several participants felt positive about the group counselling sessions because the face-to-face counselling enabled them to engage with doctors (*n* = 4). Participants highlighted that the session was convincing and that they were confident in the messages delivered as the counselling was conducted by a doctor (*n* = 2). On the other hand, the delivery of text messages and reinforcement videos was indicated as poor by the data collectors. The participants were incompetent and uncomfortable with the use of the text messenger and WhatsApp applications. 

### 3.4. Acceptability of Low Salt and High-Nitrate Vegetable Intervention

**Figure 4:**
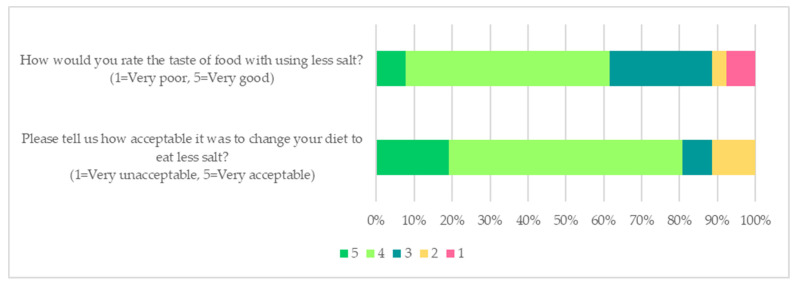
The self-rated acceptability of low salt diet.

Forty participants were randomised into the low salt interventions (low salt group *n* = 23; combined high-nitrate vegetable consumption plus low salt group *n* = 17), and the low salt acceptability data assessed at the end of the study were available for 26 participants. Most participants (81%, 21/26) found that changing to a low salt diet was either acceptable or very acceptable, and more than half of the participants (62%, 16/26) rated food with less salt as tasting either good or very good (Figure 4). Twenty participants in the low salt intervention (77%, 20/26) said they had used the salt measuring spoon and reported that it was rather easy to use (Supplementary Material, Table S5).

The reasons provided by six participants for not using the measuring spoon provided were related to: (1) personal preference, they were more comfortable in using their own measurement spoon as they claimed it to be more convenient and easier to keep; (2) family’s food habits, where the participant was either not the one who cooked in the household or meals were prepared according to the family’s palate; and (3) the perceived lack of benefit, where the participant did not see any use for the spoon.

Overall, the low salt and high-nitrate vegetable interventions were well accepted by the participants and all participants indicated that they would continue to follow the dietary recommendations. Most participants (80%, 33/41) indicated that they would recommend similar interventions to their family and friends.

## 4. Discussion

The current study was designed to examine the feasibility and acceptability of the recruitment, retention, data collection procedures, outcomes measures and intervention strategies of a dietary and behaviour intervention to reduce salt and increase high-nitrate vegetable intake. The high number of participants who were screened (*n* = 699) in a short time (about four months) demonstrated the advantage of using the SEACO database to identify and recruit potential participants. Whilst we met more than 60% (74/120) of our target sample size of 120 participants, recruitment ended prematurely as a result of the COVID-19 pandemic. The sample size of 74 would have provided the power of around 80% to detect a small effect size between 0.1 and 0.3 [56] if the trial had not been impacted by the COVID-19 pandemic. Other obstacles were related to logistical challenges, such as the scheduling of the baseline assessment visits, and participants’ barriers, including lack of interest, work commitments or child-minding duties. Similar factors affecting the enrolment of older participants in dementia research have been reported [58,59,60].

The challenges relating to scheduling baseline assessments and barriers to participation could be solved by adopting more efficient recruitment methods and study procedures. Previous studies have demonstrated that conducting recruitment at a convenient location (own home or easy to reach location) can enhance recruitment [58,61]. We have shown in this feasibility study that using a face-to-face home visit as a method to invite study participants is a successful approach for recruitment and we believe this represented one of the crucial factors in motivating an individual’s participation in the study. Whilst there is no “gold standard” recruitment method, researchers are encouraged to develop tailored and study-specific frameworks to optimise recruitment strategies through the monitoring of recruitment flow and the implementation of appropriate adaptations [62].

The original study duration was extended to approximately ten months in order to account for the COVID-19 restrictions in Malaysia, and despite the significant extension, the follow-up attendance and retention rate was over 70%. This result could be attributed to the trial being embedded within SEACO’s health and demographic surveillance site. A good relationship established between SEACO data collectors and the local community through community engagement certainly facilitated the recruitment and retention of the study. Our findings support previous studies that have found the benefit of community engagement [63] in increasing the participation in Alzheimer’s disease prevention trials [64,65]. The majority of the participants indicated that they enjoyed being part of the study and wanted to keep up to date on health- and nutrition-related information. A considerable number of dropouts were not contactable, and we do not have sufficient evidence to suggest that the change in the trial protocol might have affected the retention or that the dropouts were due to difficulties in conducting follow-up appointments through telephone calls. However, our findings warrant further work to analyse the feasibility and barriers of telephone-based data collection among these groups of participants. Other reasons (no longer interested or other commitments) might have been related to time constraints or the lack of motivation.

Evidence from previous studies supports the effectiveness of nutrition education in improving nutrition knowledge [66], diet and health in older adults [61,62,67,68]. Nutrition counselling is a vital strategy for imparting nutrition education to older adults, especially among illiterate individuals [69,70]. Consistent with other studies, we showed that group counselling was the best strategy to engage with participants. The group counselling enabled a two-way conversation between the participants and medical officers, which was considered a vital component to enhancing motivation and active participation. This could be further improved by adding more infographic materials and hands-on activities in the group counselling session.

Previous studies have shown the acceptability and use of text message reminders in improving adherence for the treatment of high blood pressure [71] and health-related behaviour among hypertensive patients [72] in LMICs. In our study, the text message was also found to be a helpful reminder, a source of information and a follow-up by the majority of participants who claimed to have read the text messages. However, the utility of the text message reminders and video messages might have been affected by the limited knowledge of some participants in the use of mobile apps. A recent feasibility study conducted among community-dwelling older Malaysians has also reported the modest acceptability and retention of information delivered via electronic media [73]. Older adults may be unfamiliar with the latest electronic devices and their facets, and they often rely on younger family members to explain and access the devices. Therefore, future studies should consider the addition of training sessions on the use of technology and communication devices in order to enhance retention and engagement with the study protocol.

This study provided learning points that are useful for informing and refining future study procedures, clinical outcomes measures, intervention materials and delivery. Key actions emerging from the study findings include a review of the screening eligibility criteria for the selection of participants using the SEACO health database to ensure screening and recruitment are efficient.

The recruitment of older participants in research trials has been acknowledged as one of the common challenges encountered in many studies [58,74]. These barriers may be related to selective eligibility criteria that may exclude participants based on the presence of comorbidities, medication use and limited mobility, which are common features in this age group [58]. Similar challenges were also encountered in this study and were one of the primary reasons for ineligibility. Specifically, participants prescribed with sodium-altering and psychiatric drugs and hormonal therapies and those diagnosed with acute and chronic medical conditions were excluded as these may have affected the outcomes of the study. In future studies, the efficiency of recruitment could be improved by applying broader selection criteria, if possible.

The comparison of the socio-demographic and health-related characteristics of the participants, included vs. excluded, is not only important for improving recruitment in future studies but also for having a significant impact on the generalisability of the study findings [59]. A careful analysis of the characteristics linked with a non-response is key to designing future screening strategies. For example, we observed that excluded participants and dropouts had lower MMSE and MoCA scores. This is consistent with a previous study by Sano et al. [75], which found that those who abandoned their Alzheimer’s Disease Cooperative Study Home-Based Assessment (HBA) trial had similarly lower MMSE scores. We also observed that Malay and single participants were less likely to take part in the study. A further assessment of the barriers, motivational drivers and health-related factors is needed to inform the recruitment strategies of trials recruiting older participants.

An additional consideration emerging from the results is related to the location of the study visits and, specifically, whether visits conducted in a clinical setting would be more appropriate and well-received by participants compared to those conducted in a non-clinical environment, such as the SEACO office space. Our findings indicated several challenges faced by the research team in scheduling the baseline clinic visits. For example, visits could only be scheduled during office hours and weekdays while some of the participants were only available during weekends. Hence, we conducted some of the visits at the SEACO office during weekends to increase the number of participants, which was further improved when we fully shifted the baseline assessments to the SEACO office in order to try to increase our recruitment efforts following the start of the pandemic. These findings suggest that having flexibility in scheduling the visits both in relation to the time and location of the visits could confer great advantages in trying to match study milestones with the preferences of older participants.

Future studies should also aim to prioritise the measurement of essential outcomes to reduce the burden on participants. All of the outcome measures in this study have been commonly applied in older adults, including validated cognitive assessment tools, health and lifestyle questionnaires and functional performance tests. The baseline assessment visits were time-consuming (~60 min), but all participants were able to complete the assessments. The measurement protocols were largely feasible with minimal missing data, with the exception of the Trail Making B test as illiterate participants could not read English letters. The challenges in performing the Trail Making B test with participants with a low education have been reported previously, particularly in non-native English speakers [76,77]. Future trials should carefully select cognitive testing tools, especially in less-educated participants, and a rational selection of the outcome measures is needed to limit the burden to participants. 

Finally, our findings indicated that a revision of the information material provided to participants is warranted in a future study. The use of written educational material is often adopted to support and enhance nutritional counselling. In the present study, the information booklet was not consulted by some participants. The primary reasons for not checking the booklet were difficulties with reading the text (*n* = 2), no time to read (*n* = 1) and misplacement and inability to find the information booklet (*n* = 5). The limited engagement was also reflected by the number of participants (51%) who tried the recipes or accessed the additional material suggested in the booklet (37%). Participants who expressed dissatisfaction (not useful, difficult to understand and irrelevant) or neutral opinions about the information booklet were among those that misplaced the information booklet or did not read the booklet. This may indicate that some of the content of the booklet may have been difficult to understand, leading to a loss of interest in reading the material. We also found that the majority of the criticisms were from the high-nitrate vegetable group. Specifically, the recipes were criticised for being culturally inappropriate for the Chinese population. This important finding is in agreement with previous research suggesting that the cultural background of older adults may affect their perception of food and that traditions may also act as barriers to the adoption of healthier nutrition behaviours [78]. Potential modifications that may improve the material presented in the booklet include (1) increasing the font size of the text, (2) refining the information and recipes to ensure that the content is tailored for a range of ethnicities and (3) incorporating more images and illustrations to facilitate the understanding of the health messages.

Quantitative and qualitative data collected from both participants and data collectors provided useful learning points to inform the design of a full-scale DePEC-Nutrition trial. Various strategies were adopted in the DePEC-Nutrition trial, including group counselling, an information booklet, text messages and video messages. The findings provided valuable insights into the type of strategies that may be more feasible and acceptable and suggested key modifications to improve the feasibility and acceptability of future strategies among this target group. Limitations included not being able to conduct a full pilot study as planned due to the COVID-19 pandemic and the consequently imposed movement restrictions. While these unprecedented conditions certainly limited our ability to establish the full feasibility of the study, particularly in relation to the measurement of quantitative outcomes, the data clearly demonstrated the feasibility of the study protocols and dietary interventions. We suggest that the definitive DePEC-Nutrition trial should employ a mixed-method approach to deliver the interventions with sessions organised both live and remotely through a telephone or other digital platforms.

## 5. Conclusions

This study successfully demonstrated the feasibility and acceptability of a combined multi-modal dietary intervention aimed at modifying dietary nitrate (increase) and sodium (decrease) intake in middle-aged and older Malaysian adults with elevated blood pressure. The interventions were well-received with a satisfying completion rate and minimal missing data for the outcome measures. The present study identified some useful learning points to inform the refinements of the study procedure, clinical outcomes selection and measurements and the amendment of the intervention materials for a design and delivery of a full-scale DePEC feasibility and RCT.

## Figures and Tables

**Table 1:** The baseline characteristics of the participants.

Characteristics ^a^	Total (*n* = 74)	Low Salt (*n* = 23)	High-Nitrate Vegetable (*n* = 17)	High-Nitrate Vegetable + Low Salt (*n* = 17)	Control (*n* = 17)	*p*-Value
Age, years	61.6 ± 6.7	62.6 ± 7.2	61.9 ± 6.7	60.4 ± 7.3	61.0 ± 5.8	0.752
Ethnicity						
Malay	51 (68.9)	17 (73.9)	9 (52.9)	13 (76.5)	12 (70.6)	0.457
Chinese	23 (31.1)	6 (26.1)	8 (47.1)	4 (23.5)	5 (29.4)	
Sex: Male	31 (41.9)	7 (30.4)	9 (52.9)	9 (52.9)	6 (35.3)	0.386
Employment status						
Working, full-time/self-employed	26 (35.1)	6 (26.1)	5 (29.4)	11 (64.7)	4 (23.5)	0.075
Working, part-time	9 (12.2)	5 (21.7)	1 (5.9)	0 (0.0)	3 (17.6)	
Retired/Unemployed /Homemaker	39 (52.7)	12 (52.2)	11 (64.7)	6 (35.3)	10 (58.8)	
Highest education level						
No formal education	6 (8.1)	1 (4.3)	2 (11.8)	1 (5.9)	2 (11.8)	0.465
Primary	27 (36.5)	10 (43.5)	8 (47.1)	3 (17.6)	6 (35.3)	
Secondary	36 (48.6)	11 (47.8)	6 (35.3)	10 (58.8)	9 (52.9)	
Tertiary and others	5 (6.8)	1 (4.3)	1 (5.9)	3 (17.6)	0 (0.0)	
Marital status						
Married	62 (83.8)	18 (78.3)	14 (82.4)	16 (94.1)	14 (82.4)	0.643
Never married/Divorced/Widow/Widower	12 (16.2)	5 (21.7)	3 (17.6)	1 (5.9)	3 (17.6)	
Current smoking (Yes)	12 (16.2)	4 (17.4)	4 (23.5)	2 (11.8)	2 (11.8)	0.841
Current use of alcohol (Yes)	8 (10.8)	2 (8.7)	4 (23.5)	1 (5.9)	1 (5.9)	0.456
BMI, kg/m^2^	27.5 ± 4.6	28.3 ± 5.5	27.2 ± 4.6	27.5 ± 3.4	26.9 ± 4.4	0.806
Systolic blood pressure, mmHg	135.9 ± 14.1	135.0 ± 15.3	136.6 ± 10.5	137.5 ± 16.5	134.9 ± 13.9	0.934
Diastolic blood pressure, mmHg	80.3 ± 9.7	81.1 ± 10.6	80.8 ± 5.8	79.5 ±12.3	79.6 ± 9.5	0.940
Geriatric Depression Scale score	4.0 (2.0, 5.0)	4.0 (2.0, 5.5)	4.0 (2.0, 5.0)	3.0 (1.0, 4.0)	2.5 (2.0, 4.3)	0.227
Grip strength, mm	26.7 ± 9.5	26.2 ± 9.6	25.3 ± 9.1	29.0 ± 10.3	26.5 ± 9.3	0.698
Physical activity, MET–mins/week	2880 (710, 5415)	2960 (660, 3840)	1920 (420, 4620)	5040 (760, 8840)	3900 (1560, 10875)	0.605
Gait speed test, m/s (*n* = 67) ^b^	1.1 ± 0.3	1.1 ± 0.3	1.1 ± 0.2	1.1 ± 0.2	0.9 ± 0.3	0.122
Timed up and go, s (*n* = 70) ^b^	9.8 (8.8, 11.8)	9.6 (8.6, 11.5)	9.3 (8.6, 11.0)	11.4 (8.9, 12.1)	10.2 (9.1, 10.2)	0.283
MoCA total score	20.8 ± 4.0	20.3 ± 4.4	21.5 ± 4.0	20.6 ± 3.8	20.9 ± 3.9	0.834
Trail Making Test B, s	207 ± 86	221 ± 87	222 ± 95	194 ± 80	188 ± 83	0.518
Animal Naming (*n* = 73) ^b^	14.9 ± 4.2	15.8 ± 3.5	13.8 ± 3.5	14.1 ± 3.4	15.5 ± 5.8	0.337
AVLT Trial 8, A7 (Delayed recall)	6.7 ± 3.4	6.7 ± 2.8	5.9 ± 4.9	6.9 ± 2.6	7.3 ± 3.3	0.684

^a^ Categorical variables are expressed as *n* (%) while continuous variables are expressed as the mean ± standard deviation (SD) for the normally distributed variables and as the median (Quartile 1, Quartile 3) for the non-normally distributed variables. A statistical analysis was conducted for comparing variables among the four groups using One—way ANOVA for the normally distributed continuous variables and Kruskal–Wallis for the non-normally distributed variables. The Chi-squared test and Fisher’s exact test were conducted for comparing categorical variables among the four groups. ^b^ Where measurements were not obtained in the full set of 74 participants, the exact number of participants for the variable is stated in brackets beside the variable name.

**Table 2:** The quantitative and qualitative results regarding the acceptability of screening and recruitment.

Category	Quantitative Data	Qualitative Data-Representative Quotes from Respondents and Data Collectors
Number of visits	Appropriate: 90.7% (49/54) Too many: 5.6% (3/54) Too few: 3.7% (2/54)	“*…many respondents complained that there were too many visits to their house and calls during baseline, clinic visit, interim 1, interim 2 and end study assessments. It made them lose interest and not want to continue the project*” (Data collector 3) “*…always the same (questions) only…*” (Participant #20) “*Asking many questions difficult to answer*” (Participant #60) “*Because sometimes not free. Got other works*” (Participant #62) “*Come (to my house) too less (frequent)*” (Participant #33)
Duration of visit	Appropriate: 84.9% (45/53) Too long: 11.3% (6/53) Too short: 3.8% (2/53)	“*Time for the counselling at the clinic was too short*” (Participant #51) “*A home/clinic visit used up to 2 h and too many assessments, questionnaires and samples to be done, it is too tiring for the elderly*” (Data collector 5)
Location of the clinic	Convenient: 96.2% (50/52) Inconvenient: 3.8% (2/52)	“*The differences are while at their home, they might feel more comfortable since we are at their house. Compared to in the clinic, the situation might be more stressful since there are also other patients in the clinic. They also needed to move from one station to another, where the distance was quite far because we used two different buildings*” (Data collector 2)

**Table 3:** Summary of feasibility and acceptability of the intervention strategies.

Intervention Strategy	Engagement with Intervention	Perceived Usefulness of Intervention Strategies (Qualitative Data)	
		Theme Generated	Representative Quote’s
Group counselling sessions	All participants from the intervention groups (*n* = 47) attended the group counselling sessions	Provide information on health and nutrition As a reminder Engagement with doctor Moderately useful	“*It can give awareness to us and can increase our knowledge about health*” (Participant #4) “*For uncle, it is good for people aged 45, 50, 60, to remind about healthy food habit*” (Participant #64) “*Can face-to-face ask doctor question. If there is doctor it will be more confident*” (Participant #18) “*I can’t change my diet immediately, it takes time, the explanation from the doctor was good*” (Participant #070)
Bi-weekly text message	Interim 1: 49% (22/45) claimed they read the text messages Interim 2: 69% (31/45) claimed they read the text messages End study: 80% (33/41) claimed they read the text messages	A reminder Provide information and knowledge Follow up Moderately useful Poor delivery	“*Because it reminds me*” (Participant #3, Interim 1) “*Give advice and show things that healthy*” (Participant #26, Interim 2) “*I didn’t mean that that (message) not help, based on what I had read, yes if really to be very helpful, if I was the one who cooked*” (Participant #40, Interim 1) “*Elderly not familiar with the technology even on using WhatsApp or open the messages, they also claimed on not received any messages or videos while our record shown they have received*” (Data collector 6)
Reinforcement video	Interim 1: Reinforcement delivered through home visit (*n* = 18); the video was sent to 85% of participants (23/27) Interim 2: The video was sent to 74% (31/42) of participants; reinforcement delivered by telephone call (*n* = 11)	Poor delivery	“*Only minority has watched the videos/messages (even mostly claimed that they have watched) respondents not familiar with using technology. Respondents claimed that they did not receive videos/messages, the records show they have received it*” (Data collector 5)
	Interim 3: The video was sent to 86% of participants (55/64); reinforcement delivered by phone call (*n* = 7) or posted compact discs (*n* = 2) End study: 85% (35/41) claimed they had watched the video		
Information booklet	At interim 2, at least 14 (31%) of 45 participants who completed the assessment admitted that they did not read the information booklet At the end of the study, only about half of the participants (51%; 21/41) claimed that they tried the recipes in the booklet provided	A guide A reminder Provide information Food choice	“*Reduce salt in cooking according to recipe book*” (Participant #29, Salt, Malay, Interim 2) “*Always remind me to reduce salt in cooking*” (Participant #42, combined, Malay, Interim 1) “*I can know the type, type of food that rich in nitrate. Sometimes we don’t know that source where it comes from. Other than that, it very for knowledge only. Like change this*” (Participant #18, Nitrate, Chinese, Interim 1) “*It provides the information in the type of vegetables and we know which one to choose*” (Participant #18, Nitrate, Chinese, Interim 2)

## Data Availability

The data are not publicly available due to privacy concerns.

## Supplementary Materials

The following are available online at https://www.mdpi.com/article/10.3390/nu14030430/s1: Table S1, reasons for exclusion from the study (*n* = 474); Table S2, reasons for the rejection of study participation (*n* = 58); Table S3, the characteristics of consented and rejected to participate in the DePEC intervention; Table S4, a summary table of the data collector feedback on data collection; Table S5, participant feedback on the salt measuring spoon. DePEC-Nutrition Resources.

## Appendix A

**Table A1:** Eligibility criteria for trial participants.

Inclusion criteria	Aged 50–75 years Live within 5 km radius from Health Clinic Sungai Segamat Systolic blood pressure 120–159 mmHg or diastolic blood pressure 80–99 mmHg BMI > 18.5 kg/m^2^
Exclusion criteria	Inability to consent Severe visual or hearing impairment Have functional impairment (Katz Index of Activities of Daily Living score < 6) Limited mobility due to any reason Current participation in other clinical studies Patients on any therapeutic diet, such as weight loss treatments or gluten-free diet Vegetarianism (likely to have a high intake of vegetables): Those who are strict vegetarians in their regular diet. Since Chinese and Indian participants are more likely to follow a vegetarian or semi-vegetarian diet, in order to maintain representativeness across ethnic groups included in the study, only participants on a strictly vegetarian diet (i.e., vegans) will be excluded from the study History of active cancer and any diagnosis of malignant cancer in the last 5 years History of excessive alcohol intake (>21 units of alcohol per week) Diagnosis of acute and chronic medical conditions interfering with the study outcomes, such as systemic infections, severe liver and kidney diseases, inflammatory bowel diseases, coronary heart diseases, cerebrovascular diseases or diabetes on insulin therapy Major surgical operations (recent/planned) interfering with the study outcomes Current diagnosis of moderate or severe depression, or other serious mental or brain disorders currently treated with psychiatric drugs (antidepressants, sedatives, antipsychotics) Subjects on sodium-altering drugs (angiotensin-converting-enzyme (ACE) inhibitors, diuretics), organic nitrates, proton-pump inhibitors, corticosteroids (systemic use only) Subjects on hormonal therapies (oestrogens, thyroxin and progesterone), antihypertensive (Ca++ channel blockers, beta-blockers) statins, oral anti-diabetic medications and any other antidyslipidemia agent Planning to move house in the next one year MMSE score ≤ 18

**Table A2:** The outcome measures for the DePEC-Nutrition feasibility study.

Outcome Measures	Description of Measures Used
Primary outcomes	
Screening, recruitment, follow-up rate and retention	Number screened: The number of people assessed for eligibility using inclusion/exclusion criteria. Eligibility rate: The number of people screened for eligibility by the number who met inclusion criteria. Reason for exclusion: Reason for ineligibility recorded by data collectors as in the field notes. Consented rate: The number of people who are eligible by the number who consented to participate in the study. Reason for declining: Reason provided by participants who declined to participate was recorded by data collectors in the field notes.
	Follow-up rate: The number of participants who were assessed during interim follow-ups. Retention rate: The number of participants who remained in the study. Reason for dropped out: Reason provided by participants for withdrawing recorded by data collectors in the field notes. Motivation to participation: Reason provided by participants for participation in the study during the end of study evaluation.
Suitability and acceptability of data collection procedures and outcomes measures	Self-rating appropriateness and suitability of intervention timing and location Feedback from data collectors on home visits and clinic visits Missing data from questionnaires: Number of participants with invalid data for specific instruments. Number of participants provided biological sample and physical measurements (blood pressure and anthropometric measurement) Participants’ self-rating acceptability of providing biological samples and physical measurement Data collectors’ feedback on the acceptability of collecting biological samples and physical measurement
Feasibility and acceptability of the intervention strategies	Engagement with the intervention: Meaningful engagement was determined by whether the information booklet provided was read by the participants. For the text messages and reinforcement video, engagement was determined by whether the participants opened, read and responded to the text messages and video. Participants self-rating of usefulness of interventions strategies or materials
Low salt and nitrate intervention	Participant self-rated acceptability of low salt diet Participant self-reported use of salt measuring spoon and easiness of use Number of participants interviewed who will continue to follow the recommended diet Number of participants interviewed who will recommend participation in a similar study to their family and friends
Outcomes for a definitive trial *	
Demographic and medical history	Demographic, medical and medication history
Health and lifestyle	Global Physical Activity Questionnaire (GPAQ), Global Activity Limitation Indicator (GALI), smoking and alcohol use, Geriatric Depression Scale (GDS) (short form)
Cognitive assessment	Montreal Cognitive Assessment (MoCA) test, Auditory Verbal Learning Test (AVLT), Trail Making Test Part B, Animal Naming Test
Blood pressure	Three consecutive measurements of resting blood pressure readings in a sitting position using OMRON automated monitor (OMRON HEM 907, OMRON Healthcare, Milton Keynes, UK)
Anthropometry and body composition	Height (m), weight (kg), BMI (kg/m^2^) and body composition were measured using a portable bioelectrical impedance analysis (BIA) scale (Tanita DC-430MA Body Composition Analyzer, Tanita Corporation, Japan) with a 0.1 kg precision
Functional performance	Hand grip strength dynamometer, 4-metre gait speed test, Timed Up and Go (TUG) test
Dietary assessment	Food Frequency Questionnaire, 24-h diet recall
Biological sample collection	15 mL whole venous blood, 24-h urine sample, spot urine sample, whole saliva collected using passive drool technique, salivary strips and dried blood spot

* These outcomes were collected at baseline only due to the COVID-19 pandemic and require further pilot testing in a definitive trial.
