# Supplementary material for: Feasibility and Acceptability of a Dietary Intervention to Reduce Salt Intake and Increase High-Nitrate Vegetable Consumption in Malaysian Middle-Aged and Older Adults with Elevated Blood Pressure: Findings from the DePEC-Nutrition Trial
Source: Nutrients. 2022 Jan 19;14(3):430. doi: 10.3390/nu14030430 (PMC8839221; doi:10.3390/nu14030430)
Supplement: Supplementary file 1 [file nutrients-14-00430-s001.zip › nutrients-1463992-supplementary.pdf]

Table S1: Reason for exclusion from the study (n=474)

Exclusion category	n	%
Medication	150	31.6
Acute and chronic medical condition	74	15.6
On Insulin therapy	51	10.8
Normal BP (SBP <120 mmHg AND DBP <80 mmHg)	45	9.5
Limited mobility	32	6.8
Major surgical operations	25	5.3
SBP≥ 160mmHg OR DBP≥100 mmHg	24	5.1
Planning to move house in the next one year	15	3.2
MMSE score ≤18	15	3.2
Visual or hearing impairment	10	2.1
On therapeutic or vegetarian diet	10	2.1
Depression, brain or mental disorder	7	1.5
Functional impairment (Katz Score <6)	4	0.8
Inability to consent	3	0.6
Excessive alcohol intake	3	0.6
Active and any diagnosis of cancer in 5 years	3	0.6
Current participation in other clinical studies	1	0.2
BMI ≤ 18.5 kg/m^2	1	0.2
Age >75 years	1	0.2

Table S2: Reason for rejection to study participation (n=58)

Category for rejection	n	%
Not interested in the study	36	62.1
Not a suitable time (Grief / in the period of mourning/ busy with other work)	8	13.8
Too frequent visits from SEACO	7	12.1
Complete avoidance	4	6.9
Appointments scheduled but eventually refused with no reason	2	3.4
Unwilling to go clinic visit	1	1.7

Table S3: Characteristics of consented and rejected to participate in the DePEC intervention

Characteristics	Recruitment			Retention		
	Consented (n=97)	Rejected (n=58)	p-value	Retained (n=54)	Dropped (n=20)	p-value
**Age, years**	61 ± 7	59 ± 7	0.093	62 ± 7	61 ± 7	0.461
**Ethnicity**						
Malay	67 (57.3)	50 (42.7)	0.031	34 (66.7)	17 (33.3)	0.069
Chinese	29 (78.4)	8 (21.6)		20 (87.0)	3 (13.0)	
Indian	1 (100.0)	0 (0.0)		0 (0.0)	0 (0.0)	
**Sex: Male**	40 (70.2)	17 (29.8)	0.169	24 (77.4)	7 (22.6)	0.465
**Employment Status**						
Working, full time/self employed	31 (59.6)	21 (40.4)	0.909	20 (76.9)	6 (23.1)	0.443
Working, part-time	4 (66.7)	2 (33.3)		5 (55.6)	4 (44.4)	
Retired/Unemployed/homemaker	62 (63.9)	35 (36.1)		29 (74.4)	10 (25.6)	
**Highest education level**						
No formal education	7 (77.8)	2 (22.2)	0.735	3 (50.0)	3 (50.0)	0.348
Primary	34 (59.6)	23 (40.4)		19 (70.4)	8 (29.6)	
Secondary	48 (61.5)	30 (38.5)		27 (75.0)	9 (25.0)	
Tertiary and others	8 (72.7)	3 (27.3)		5 (100.0)	0 (0.0)	
**Marital status**						
Married	81 (67.5)	39 (32.5)	0.019	46 (74.2)	16 (25.8)	0.724
Never married/divorced/ widow/widower	16 (45.7)	19 (54.3)		8 (66.7)	4 (33.3)	
**Current smoker**	15 (71.4)	6 (28.6)	0.470	10 (83.3)	2 (16.7)	0.377
**Alcohol consumer**	10 (100.0)	0 (0.0)	0.014	7 (13.0)	1 (5.0)	0.435
**BMI, kg/m^2**	28.2 ± 4.4	27.6 ± 4.9	0.425	27.2 ± 4.2	28.4 ± 5.6	0.343
**Systolic blood pressure (mmHg)**	136 ± 10	135 ± 13	0.344	135.8 ± 12.3	136.3 ± 18.4	0.911
**Diastolic blood pressure (mmHg)**	78 ± 7	76 ± 9	0.140	79.9 ± 9.1	81.5 ± 11.3	0.535
**MMSE score**	25.4 ± 2.6	24.7 ± 2.6	0.093	25.7 ± 2.6	24.2 ± 2.7	0.022
Normal (MMSE score ≥24)	77 (67.0)	38 (33.0)	0.061	46 (79.3)	12 (20.7)	0.019
Mild (MMSE score 19-23)	20 (50.0)	20 (50.0)		8 (50.0)	8 (50.0)	
**Geriatric Depression Scale score**	-	-	-	4.0 (2.0, 5.0)	4.0 (2.0, 5.0)	0.661
**Grip strength (mm)**	-	-	-	27.1 ± 9.9	25.6 ± 8.3	0.542
**Gait speed test (m/s)**	-	-	-	1.1 (0.3)	1.0 (0.3)	0.128
**Timed up and go (s)**	-	-	-	9.8 (8.9, 11.5)	9.9 (8.6, 12.1)	0.973
**MoCA total score**	-	-	-	21.4 ± 4.0	19.1 ± 3.7	0.023
**Animal Naming**	-	-	-	14.5 ± 4.0	16.0 ± 4.4	0.183
**Trail Making Test B**	-	-	-	198.8 ± 83.8	230.0 ± 90.4	0.168
**AVLT Trial 8, A7 (Delayed recall)**	-	-	-	6.7 ± 3.5	6.7 ± 3.4	0.971

[a] Categorical variables are expressed as n (%) while continuous variables are expressed as mean ± standard deviation (SD) for normally distributed variables and median (Quartile 1, Quartile 3) for non-normally distributed variables.

[b.] Statistical analysis was conducted for comparing variables among two groups (enrolled versus rejected and retained versus dropped) using independent T-test for normally distributed continuous variables, Mann-Whitney test for non-normal distributed continuous variables and Fisher's Exact test for categorical variables.

Table S4: Summary table of data collector feedback on data collection

Theme	Subtheme	Representative Quotations
Home and clinic visit	Number	*…many respondents complain that there are too many visits to their house and calls as we conduct Baseline, Clinic Visit, Interim 1, Interim 2 and End Study Questions. It made them lose interest and did not want to continue the project. (Data collector 3)*
	Duration	• *It is too long, as in one visit there are many sections of questions (eg; MoCA, recall, health questions, physical activities) that respondent needs to answer/do. (Data collector 1)*<br>• *A home/clinic visit used up to 2 hours and too many assessments, questionnaires and samples to be done, it is too fatigue for the elderly (Data collector 5)*
	Location	• *The clinic session that need them to travel from home, and walk around the clinic for different sessions, the environment is not comfortable as the clinic is crowded with patients (Data collector 5)*<br>• *The differences are while at their home, they might feel more comfortable since we are at their house. Compared to in the clinic, the situation might be more stressful since there are also other patients in the clinic. They also needed to move from one station to another, which the distance is quite far because we used 2 different buildings (Data collector 2)*<br>• *The environment at the clinic was quite stressful for respondents compared at their homes as they need to complete many assessments at different locations. (Data collector 3)*<br>• *Respondents and DC are more comfortable at home without interruption (Data collector 5)*<br>• *The environment at the clinic was quite stressful and uncomfortable for respondents compared at their homes as they need to complete many assessments at different locations. (Data collector 6)*
Measurements	Understanding of questions/ questionnaire	• *Some of the questions are not easy to understand by the elder, sometimes they feel stress resulting in loss of interest when answering the questions (Data collector 1)*<br>• *Respondents are keen to think that the (MoCA, Animal Naming) questions as a silly question (Data collector 3)*
	Number of questions or questionnaire	• *…there are too many questions that make them quickly lose interest and patience. (Data collector 3)*<br>• *It is too long and too many questions that have caused us to take a long time for at least half an hour at their house especially for screening and home baseline. That might be because we took their recess time and made them and ourselves feel uncomfortable and also led most of them to lose interest in proceeding to the next stage. (Data collector 2)*<br>• *as the respondents have to answer many questions (MoCA, Animal Naming), it makes them lose interest and also patience. (Data collector 6)*
Experience with text message reminder	Understanding	• *No, because the message is easier to understand by the elder. (Data collector 1)*<br>• *No challenges as it is straightforward to do. (Data collector 3)*<br>• *Respondents easily understand about SMS and videos given (Data collector 4)*

Table S4: Summary table of data collector feedback on data collection *(Continued)*

Theme	Subtheme	Representative Quotations
	Challenges	• *Since our respondents are among older people, some of them are not familiar with technology and they are not able to view the video message. Even the message reminder is also difficult for some respondents. Due to that, we received multiple complaints that they did not receive any message while our record shows that the message was delivered to their number. (Data collector 2)*<br>• *… some time respondents do not understand how to read message. (Data collector 4)*<br>• *Only minority has watched the videos/ messages (even mostly claimed that they have watched) respondents not familiar with using technology. Respondents claimed that they did not receive videos/ messages, the records shown they have received it (Data collector 5)*<br>• *Elderly not familiar with the technology even on using WhatsApp or open the messages, they also claimed on not received any messages or videos while our record shown they have received (Data collector 6)*
Training/ expertise or skills required		*No because we need to catch up on everything in a short time. It's a bit difficult because we are not from a nutrition background but we need to explain a lot of things that are related to nutrition and dietary. (Data collector 1)*<br>*Nutrition knowledge (Data collector 5)*<br>*Negotiation, persuasion and communication skills (Data collector 1, 2, 3, 6)*
Experience with phone call	To reach out to participant	• *Challenges faced especially during phone interviews due to the pandemic. Most respondents might be afraid to pick up calls since there are too many scammers nowadays (Data collector 2)*<br>• *Respondents hardly approached (do not pick up phone, number not in service) (Data collector 5).*<br>• *They also keep giving excuses to avoid talking to us because we keep calling (Data collector 3). Sometimes it is also hard to make appointments through phone (Data collector 2)*
	Challenges of data collection	• *It is more difficult to explain in detail what the question is about compared to by face to face and it may lead to confusion for them. (Data collector 2)*<br>• *There are half of the respondents who do not understand the question asked by DC even though the question is repeated (Data collector 4)*<br>• *Questions are hardly explained especially to the elderly through phone (Data collector 5)*<br>• *Phone interviews were quite challenging when the respondent can't imagine or even understand the score and questions given as the choice of answer was confusing. (Data collector 3).*
	Other challenges	• *Line not clear (can't listen to what they said/ they can't listen to us). (Data collector 5)*<br>• *Sometimes they did not even hear properly as they quickly lost focus that make them did not understand the interview. Respondents also did not want to talk too long on the phone (Data collector 3*

Table S5: Participant feedback on salt measuring spoon

Theme	Subtheme	Quotes
Use	Easy to use (n=5)	*"That spoon more OK because it has the number. Easy, easy. I really use" (Participant #3, Interim 1)*<br>*"The spoon has two edge, big and small. I can use both side for my measurement. It's easy to use" (Participant #70, Interim 1) … "The measuring spoon indication was clear" (Participant #70, Interim 2)*<br>*"I use that small 5-digit spoon. It got write the number, easier for me, know how much" (Participant #48, Interim 2)*<br>*"…make measuring salt easier" (Participant #51, Interim 2)*<br>*"Measure at that salt measuring spoon then put at our spoon" (Participant #65, Interim 2)*
	Helpful (n=4)	*"Yes. My wife used it (the spoon). I saw from far she used it...Because can help to measure the salt" (Participant #6, Interim 2)*<br>*"Helpful. That spoon I used; 1 measurement 5 g can cook 3 types" (Participant #23, Interim 2).*<br>*"When we know that salt can't be too much and got measurement. As much as this only" (Participant #26, Interim 1)*<br>*"…Because previously I eat a lot of salt and sugar. So when got measuring spoon, it help me to reduce salt intake" (Participant #49, Interim 2)*
Not using	Not the one who cook (n=3)	*"…if I cook, I use, but if uncle cooks (respondent laughing) not use" (Participant #4, Interim 2)*<br>*"…then with all efforts sometimes I told my wife who is cooking, why didn't use the measuring spoon...depends, depends on the condition of that dishes" (Participant #40, Interim 2)*<br>*"…my wife prepare the meal and she is more comfortable in using her own measurement spoon" (Participant #54, Interim 1)*
	Use their own spoon (n=8)	*"Didn't use salt measuring spoon, use my own spoon" (Participant #27, Interim 2)*<br>*"I did not use, but I had a small spoon. I had a spoon to measure salt; before this I tried to measure, compared with the spoon that I had" (Participant #29, Interim 1)*<br>*"We already had that salt spoon, I think it is same with that salt spoon...Salt spoon that being sell had a salt container. Just follow the usual salt spoon" (Participant #30, Interim 1)*<br>*"For me it's useful but I did not use it (spoon)...I'm using a regular spoon only" (Participant #38, Interim 1)*<br>*"…use KFC spoon as it convenient and easy to keep" (Participant #48, interim 1)*<br>*"Hard to use the spoon as I only use a bit of salt, I prefer to use my own measurement spoon." (Participant #52, Interim 1)*<br>*"When we cook, it's kind of inconvenient to use the measuring spoon you provided for cooking as it took up space compared to the spoon we used" (Participant #67, Interim 2)*<br>*"not really use it (measuring spoon) …if use, use my own sense of taste" (Participant #64, Interim 1)*
	Family (n=1)	*I am not using the measure aunty use salt but my cooking is not salty… sometimes many children daughter in law all here" (Participant #24, Interim 1)*
	Perceived not applicable (n=1)	*"Didn't take back the spoon. Didn't add salt, add soy sauce only" (Participant #55, Interim 1)*

DePEC Resources

DePEC information booklet for reduce-salt group

DePEC – NUTRITION (SALT)

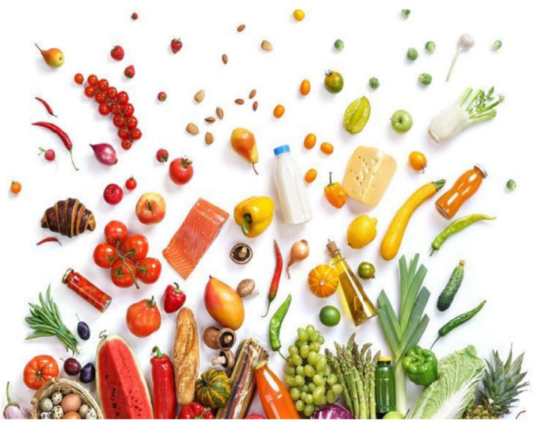

Table of Contents

	Pages
Eating healthily – why is it important?	1
Salt – What is it?	2
Salt and Health	2
Recommended Intake	3
Common Sources of Salt in Malaysia	4
How to be more salt aware?	7
Reading Food Labels	8
Salt content of common condiments used in Malaysia	10
Food Swaps	11
Common misperceptions about salt reduction	12
Salt Measuring Spoon	13
How to use the salt measuring spoon?	13
Meal suggestions low in salt	15

Eating healthily – why is it important?

Eating well means you are more likely to feel healthier, stay active for longer and protect yourself against illness. It is never too late to start eating healthily, and a healthy diet does not have to be restrictive, boring or expensive.

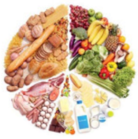

Following a healthy diet has multiple benefits, including: increased energy, improved sleep quality, improved mood and mental wellbeing as well as lowering the risk of chronic health conditions.

In particular, the food we consume is directly related to many of the risk factors for cardiovascular disease – including heart disease, heart failure and stroke - which is one of the main causes of death and disability worldwide. For example, saturated and trans fats increase blood cholesterol which increases your chance of having a heart attack, while polyunsaturated and monounsaturated fats lower the risk of heart attacks. Eating too much salt raises blood pressure, putting extra strain on the cardiovascular system and increasing risk of heart disease, whereas eating more fruit and vegetables can have a preventative effect, as they contain powerful nutrients such as dietary nitrates, which can improve blood pressure and heart health. Furthermore, evidence shows a potential link between the foods we eat and brain processes, such as our thinking or cognitive skills, and as a result by eating more healthily, it can help to reduce the risk of developing dementia.

In this leaflet, we will provide information on one dietary component – salt. Research has shown that by **reducing** the amount of salt you eat, you can improve your heart health, especially your blood pressure. This information leaflet will give advice on common sources and recommended intakes, provide practical tips as well as recipes for you to try at home. Take time to read through the material and try to make some changes to your diet based on the advice we have provided.

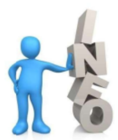

Salt – What is it?

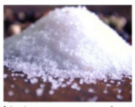

Salt is the everyday term we use for a chemical compound called sodium chloride.

Although it is not a requirement, some manufacturers may still quote the sodium content of foods on their labels. It is important to know that **each gram of sodium translates to 2.5 grams** of salt, and to take this into account when looking at your everyday salt consumption.

Many everyday foods are not obviously salty, but they can contain high amounts of 'hidden salt'. While we may add salt to our food during cooking or just before we eat it, a large amount of the salt we eat is already added to the food we buy. Most of this comes from salt added to foods like processed meat products, snacks (such as crackers and biscuits), soups and sauces, including soy sauce.

Salt and Health

Reducing salt intake has been identified as one of the most cost-effective measures countries can take to improve the health of their population. While sodium is essential for muscle and cell function, eating too much salt raises blood pressure and increases the risk of developing heart disease and having a stroke.

High blood pressure puts added force against the walls of your arteries (the blood vessels that carry blood around the body). Over time, this extra pressure can damage the arteries, which makes them more likely to become narrowed and hardened by fatty deposits. When this happens it can increase the risk of having angina or a heart attack.

Research has also shown that having high blood pressure can also increase your risk of developing dementia, in particular vascular dementia. Uncontrolled high blood pressure can cause problems by damaging the blood vessels in your brain. Over time, this raises the risk of a blood vessel becoming blocked or bursting. If a blood vessel cannot carry energy and oxygen to a part of the brain properly, some cells in the brain may be

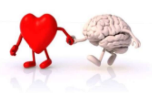

damaged. This damage can sometimes affect a person's memory, thinking, or language skills.

Recommended Intake

Average salt intake among Malaysians is around 7.15 grams per day, exceeding the World Health Organisation (WHO) recommendations.

WHO recommends a reduction **to less than 2 grams of sodium per day**, which is equivalent to **5 grams of salt per day** - this is less than one teaspoon of salt per day.

Common Sources of Salt in Malaysia

The most common foods that are high in salt in Malaysia, with low salt alternatives, have been listed below. Increase the number of lower salt foods in your diet and limit high salt options within the same food group. ***Please note:*** *We have included foods that may be consumed by a range of cultures and ethnic groups. Please refer to the foods that are inclusive of your own cultural beliefs.*

Common foods HIGH in salt	LOW salt options	Salt Reduction GOALS
	**Condiments and Spices**	
Kicap cair* (Light soy sauce)**<br>Kicap pekat* (Dark soy sauce)**<br>***Sambal belacan* (Shrimp paste)**<br>***Sos tiram* (Oyster sauce)**<br>***Sos cili/ tomato* (Chilli/ tomato ketchup)**<br>***Budu* (Anchovies paste)**<br>**Mayonnaise / Salad dressing**<br>**Monosodium Glutamate**<br>***Tempoyak* (fermented durian)**<br>***Taucu* (fermented soybean paste)**<br>**Instant seasoning powder**	All natural condiments, herbs and spices (such as cloves, cinnamon, anise seeds, cumin seeds, turmeric, *asam gelugor*, cardamom, dried chilli, thyme, basil, ginger, curry powder)<br>Reduced salt soy sauce<br>Tomato puree<br>Lower sodium substitutes such as potassium salt	Replace the high salt condiments with more low salt options to add flavour to meals. E.g. you could choose reduced salt soy sauce instead of the standard varieties.<br><br>Reduce the number of times you use the high salt condiments when cooking e.g. if you use every day, try using them only 3-4 times a week.<br><br>Measure - use measuring spoons when cooking e.g. if usually add 4 tablespoons of soy sauce to a meal, try gradually reducing the number of tablespoons you add over a few weeks. Your taste buds will adapt to the lower salt taste
	**Rice and Carbohydrate Sources**	
Nasi goreng* (Fried rice)**<br>Nasi lemak* (Rice cooked in coconut milk, garnished with fried anchovies, peanut, egg, cucumber)**	Rice or noodles, plain, boiled<br>Bread, white or wholemeal<br>Biscuit, soda/plain or low salt, whole-wheat crackers	Reduce the consumption of salt while preparing or cooking these foods at home. You can do this by using measuring spoons to measure the amount of salt you add to foods/meals when cooking e.g. if you normally

Common foods HIGH in salt	LOW salt options	Salt Reduction GOALS
	**Meat and Meat Products**	
Sup daging lembu* (Mutton soup)**<br>Ayam goreng berempah* (Spicy fried chicken)**<br>**Chicken, fried, fast food franchise**<br>**Chicken curry, canned**<br>**Chicken broth cubes (pati ayam)**<br>**Beef burger with cheese**<br>**Beef *rendang*, canned**<br>**Mutton curry, canned**<br>**Meat extract (Bovril / Marmite)**	Choose fresh, unprocessed chicken, beef or mutton when preparing meat based meals at home	Reduce the consumption of salt while preparing and/or cooking these foods at home.<br><br>Replace salt with low salt condiments, herbs and spices to add flavour to vegetables when cooking.
	**Fish**	
**High salt content:**<br>***Ikan masak sambal* (Fried fish with sauce)**<br>***Bebola kek* (Fish ball)**<br>**Fish, dried, salted**<br>**Fish Sauce**<br>**Anchovy, dried, without head and entrails**<br>**Sardine, canned**<br>**Shrimp, fermented (*Cencaluk*)**<br>**Shrimp paste (*Belacan*)**<br><br>**Moderate salt content:**<br>**Fish Crackers, fried**<br>**Prawn, salted, dried**<br>**Prawn crackers**<br>**Prawn paste (*Hay-Ko*)**	Choose fresh, unprocessed fish and prawns when preparing fish based meals at home	Reduce the consumption of salt while preparing and/or cooking these foods at home. Be aware of fish sauces as they can have a high salt content.<br><br>Replace salt with low salt condiments, herbs and spices to add flavour to vegetables when cooking.

How to be more salt aware?

In this research study, we hope that you will be able to reduce the amount of salt you eat using the information provided in this leaflet. During the study, our researchers will take some measurements to look at sodium levels in your body. This will help us to see whether a reduction in salt intake has been achieved.

Here are a few practical tips to help you reduce the level of salt in your diet:

❖ Reduce the amount of salt added in cooking and the addition of other flavour enhancers such as monosodium glutamate (MSG), sauces (such as soy sauce, oyster sauce, fish sauce, tomato sauce) and flavouring cubes. Avoid using table salt or adding sauces while enjoying the food.

❖ Enhance the flavour of food using natural herbs and condiments such as garlic, onion, curry spices, white pepper, lemon grass, vinegar and lemon.

Here are some examples of herbs and spices which can enhance the flavour of different foods:

- Basil: Use with beef, chicken and most vegetables
- Curry: Use with beef, chicken, fish, green beans, carrots and in marinades
- Ginger: Use with beef, chicken, green beans, cauliflower and egg plant
- Thyme: Use with beef, chicken, fish, green beans, beets and carrots

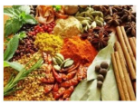

❖ Learn to enjoy natural flavour of foods without salt

❖ Reduce intake of salty foods such as salted fish, salted eggs, salted vegetables, high-sodium snacks (such as potato crisps and chips) and processed foods (such as sausages, chicken nuggets, meatballs and burgers / burger patty)

❖ Choose fresh fruits and vegetables instead of preserved and processed types. Reduce/avoid using sour plum powder, mayonnaise and salad dressing when enjoying fruits and vegetables

❖ Soak preserved foods such as dried anchovies in water to reduce sodium content

❖ Choose low sodium breakfast cereal e.g. porridge, wheat biscuits or bran flakes

❖ Drain the water from the can before consuming canned product because it is high in salt, which is used to preserve the food

❖ Eat out smartly. Eating out while controlling your sodium intake is a challenge because the sodium content of a food served is always unknown and often is higher than expectation. Therefore, it is advisable to try not eating out too often. Limit fast food consumption and request for low salt and less sauces dishes or no MSG added meals when eating out

Here are some eating-out tips for reducing sodium intake:

- Avoid adding extra salt or sauces to foods such as tomato, chilli and soy sauce
- Choose more freshly prepared options rather than processed foods like sausages, bacon and hot dog as these are likely to be high in salt
- Choose steamed vegetables as a side dish rather than French fries, for example

Reading Food Labels

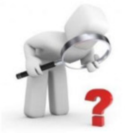

Have a look at the sodium content of a food in the Nutrition Information Panel (NIP). This is a table on the food label which lists all the major nutrients contained in the product. Compare the sodium content with other available brands of the same product and choose the ones with the lower sodium content. Try to choose brands with "low" or "lower" salt claims on the label, if available.

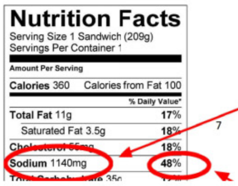

This is an example of a Nutrition Information Panel.

You will see that in 209g (1 serving) of this food product, there is 1140mg (1.14g) of sodium, or 2.85g of salt (1.14g sodium x 2.5).

This product is HIGH in salt, containing more than half of the recommended daily limit (5g).

Also, look out for the following ingredients on the food label which contain sodium:

- Monosodium glutamate (MSG)
- Sodium nitrate
- Sodium benzoate

Salt content of common condiments used in Malaysia

Below are some examples of condiments used in Malaysia with their estimated salt content per tablespoon. These have been labelled use in moderation (to consume every so often), or use sparingly (to consume in small amounts, less often).

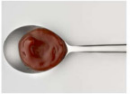

Tomato Ketchup
0.3g salt in one tablespoon serving.
One table spoon contributes towards 6% of your recommended daily salt intake of 5g.
Use in moderation

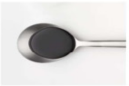

Soy Sauce
2.75g salt in one tablespoon serving.
One tablespoon contributes towards 55% of your recommended daily salt intake of 5g.
Use sparingly

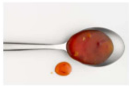

Chilli / Hot sauce
0.6g salt in one tablespoon serving.
One tablespoon contributes towards 12% of your recommended daily salt intake of 5g.
Use in moderation

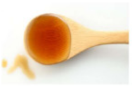

Fish sauce
2.65g salt in one tablespoon serving.
One tablespoon contributes towards 53% of your recommended daily salt intake of 5g.
Use sparingly

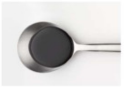

Oyster sauce
1.0g salt in one tablespoon serving
One tablespoon contributes towards 20% of your recommended daily salt intake of 5g.
Use sparingly

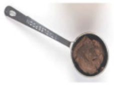

Anchovy paste
2.7g salt in one tablespoon serving
One tablespoon contributes towards 54% of your recommended daily salt intake of 5g.
Use sparingly

Food Swaps

Here are some simple food swaps you can try in order to reduce your salt intake:

HIGH SALT		LOW SALT
Fried/Curry Chicken	→	Roasted/grilled Chicken
Salted Nuts	→	Unsalted Nuts
Cream Cracker	→	Low-Salt Wholewheat Cracker
Cooked/fried vegetables	→	Fresh/raw vegetables (*ulam-ulaman*)
Fried fish	→	Steamed/grilled fish

Common misperceptions about salt reduction

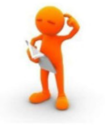

"On a hot and humid day when you sweat, you need more salt in the diet"

There is little salt lost through sweat so there is no need for extra salt even on a hot and humid day, although it is important to drink a lot of water

"Sea salt is not 'better' than manufactured salt simply because it is 'natural'"

Regardless of the source of salt, it is the sodium in salt that causes bad health outcomes

"Salt added during cooking is the main source of salt intake"

In many countries, about 80% of salt in the diet actually comes from processed foods or food eaten outside the home, for example in restaurants

"Food requires salt to have appealing flavour"

It takes some time for a person's taste buds to adjust, but once they get used to less salt, one is more likely to enjoy food and notice a broader range of flavours

"Food has no flavour without salt"

Whilst food will initially taste different without salt, taste buds soon become accustomed to less salt and you are more likely to enjoy food with less salt, and more flavour

"Foods high in salt taste salty"

Some foods that are high in salt don't taste very salty because sometimes they are mixed with other things like sugars that mask the taste. It is important to read food labels to find out sodium levels

"Only old people need to worry about how much salt they eat"

Eating too much salt can raise blood pressure at any age

"Reducing salt could be bad for my health"

It's very difficult to eat too little salt since there are so many everyday foods containing salt

Salt Measuring Spoon

We have provided you with a salt measuring spoon to help you measure the amount of salt that is recommended for daily consumption by WHO (5g per day).

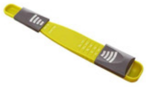

As mentioned, approximately 80% of salt consumed comes from **processed foods.** It is important to take this into consideration in relation to the total 5g daily intake, as this will include all **salt added during cooking in the home, salt already added during processing or added in preparation of meals when eating out in a restaurant or takeaway.** Therefore, it is important to read food labels or ask staff in restaurants to find out about salt content of foods.

How to use the salt measuring spoon?

1. Transfer the salt you have at home into a container that is easily accessible, e.g. a container that has a wide opening
2. Adjust the measuring spoon to the 5g setting. This is the **total amount** of salt one person can have in one day. Alternatively, by selecting the 1g setting on the spoon, you can use **5 x 1g portions per day**. From the salt container, scoop salt using the spoon and level off.
3. Add the salt to meals when cooking to your desired taste, keeping within the 5g daily allowance.

When cooking for more than one person within the household, simply multiply the daily limit by the number of people, e.g. if cooking meals for a family of 3, the total daily allowance of salt would be 15g.

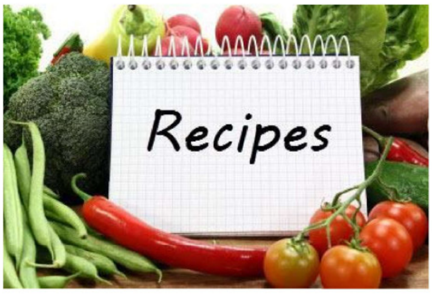

Meal suggestions low in salt

Here are some examples of low salt meals that you can prepare at home:

Vegetable *Mee Goreng* (Fried Noodles) (Serves 3-4)

Ingredients:

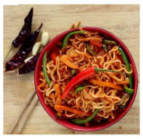

3 tablespoons cooking oil e.g palm oil
2 cloves garlic finely minced
3 pieces dried bean curd cut into small pieces
1 potato boiled, peeled, and sliced into wedges
1 big tomato cut into wedges
A handful fresh bean sprouts
1 lb fresh yellow noodles rinsed and drained
2 red chillies for garnishing, chopped
2 shallots for garnishing, chopped
1 lime cut into wedges

Sauce:
2 tablespoon *reduced salt* soy sauce
3 tablespoon tomato puree

Instructions:

1. Heat up a wok and pour in the cooking oil. Add the garlic and stir fry until aromatic then add the bean curd and potato and tomato wedges. Do a few quick stirs and then add the yellow noodles and the sauce and continue stirring until everything is well combined.
2. Add in the bean sprouts and quick stir for another 1 minute. Turn off the heat, dish out, garnish with the red chili and shallots. Squeeze some lime juice over the noodles before eating.

Spiced Rice with Chicken (Serves 4-6)

Ingredients:

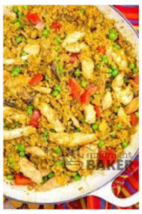

900g chicken
1 clove garlic
5cm piece of ginger
2 red chillies
1 tbsp poppy seeds
10 cashew nuts, plain, unsalted
10 almonds
2 tbsp ghee
5 cloves
5cm cinnamon stick
1 cup shallots, sliced
1 tsp curry powder
1/2 cup yoghurt, plain
1-2 cups coconut milk
400g rice

Instructions:

1. Cut chicken into pieces. Grind together garlic, ginger, chillies, poppy seeds, cashew nuts and almonds.
2. Heat ghee and fry cloves, cinnamon stick and shallots. Add chicken pieces, ground ingredients and curry powder. Stir to mix and cook covered for 10 minutes. Add yoghurt and simmer till chicken is tender and mixture is thick.
3. Add coconut milk to the rice and cook. When rice has absorbed all the coconut milk make a well in the centre and place the chicken mixture in the centre. Cover and allow the rice to finish cooking over a very low heat. Serve.

Hot and Sour Malay Fish Curry (Serves 4)

Ingredients:

2 tablespoon of cooking oil e.g. palm oil
1 cm galangal root, bruised
3-4 slices *asam gelugur* or 1 ½ heaped tablespoons tamarind pulp soaked in 4 tablespoons water, mashed and strained to obtain juice
625ml water
6 thick fresh fish fillets (about 750g)
4 sprigs laksa leaves (*daun kesum*), minced
3 tablespoons thick coconut milk
1 teaspoon sugar
1 tablespoon pineapple achar

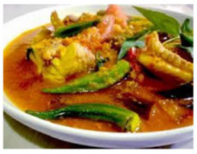

Spice paste:
2 candlenuts, roughly chopped
10 chillies, cut into lengths
10 shallots, peeled and halved
4 cloves of garlic, peeled and halved
½ teaspoon turmeric
250 ml water

Instructions:

1. Grind the spice paste ingredients to a paste in a mortar or blender, adding a little oil if necessary to keep the blades turning.
2. Heat the oil in a pot over a medium heat and stir fry the spice paste and galangal for 5 minutes until fragrant. Add the *asam gelugur* or tamarind juice and ½ cup (125ml) water, and cook for 5 minutes.
3. Add the rest of the water and bring to a boil. Then add the fish, laksa leaves, coconut milk and sugar. Simmer, uncovered, for another 5-10 minutes until the oil separates from the coconut milk. Serve with freshly cooked white rice.

Chicken *Rendang* (Serves 4)

Ingredients:

220g boneless chicken breast (raw), cut into bite size pieces

Blend the following ingredients until fine:

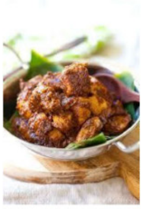

3 shallots, chopped
4 cloves garlic
3 whole fresh red chilies
1 inch ginger
2 sticks lemongrass
1 inch galangal
1 inch ground turmeric
15 cm x 1 pc turmeric leaves, slice finely
5 cm x 3 pcs kaffir lime leaves, slice finely
2 tablespoons cooking oil e.g palm oil
1⁄2 cup water

Instructions:

1. Heat oil in a heavy bottom pan or a pot. Sauté the blended spices for a minute or until fragrant.
2. Put the chicken and water in the pan or pot, and stir over medium heat and bring to a boil.
3. Reduce heat to a simmer and cook until the gravy thickens. Add turmeric leaves and kaffir lime leaves. Continue to cook until the meat becomes tender and the gravy reduced to a desired thickness.
4. Serve hot in individualized portion during meal time.

Lentil and Vegetable Curry (Serves 4)

Ingredients:

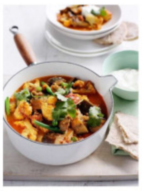

Olive oil spray or 1 tablespoon cooking oil e.g palm oil
1 large onion, thinly sliced
200g mushrooms, halved
2 garlic cloves, crushed
400g eggplant, cut into 2.5 cm dice
1-2 tablespoons korma curry paste
600g cauliflower, trimmed, cut into small florets
1 x 400g can no added salt tomatoes
1 cup reduced salt vegetable or chicken stock or water
1 x 400g can brown lentils, rinsed and drained
150g green beans, topped, halved
1/2 cup reduced fat evaporated milk
1/2 cup chopped flat leaf parsley or coriander
4 small wholemeal pita breads and 4 tablespoons reduced fat natural yoghurt, to serve

Instructions:

1. Heat a large, deep non-stick frying pan or saucepan over medium heat. Spray with oil. Add onion and cook, stirring often, for 3-4 minutes or until the onion is soft and light golden. Remove to a plate. Spray pan lightly again with oil. Add the mushrooms and garlic, cook, stirring often for 2-3 minutes or until mushrooms are lightly browned. Remove to the plate with the onions.
2. Spray pan lightly again with oil. Add eggplant and cook, stirring often for 3-4 minutes or until just softened. Add 2 teaspoons of water to create steam if the eggplant is sticking. Return the onions, mushrooms and garlic to the pan. Add curry paste, stir to coat, cook for 1 minute.
3. Stir in cauliflower, tomatoes, and stock or water. Bring to boil. Reduce the heat to low and simmer, covered for 10-15 minutes. Add lentils and beans and simmer, uncovered for a further 5-10 minutes or until beans are just cooked through. Stir through the evaporated milk and parsley or coriander.

4. Serve with pita bread and yoghurt if desired.

Tip: You can replace eggplant with sweet potato, pumpkin or regular potatoes.

Further Reading

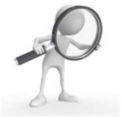

We have provided some further reading below to supplement the information described in this leaflet. These webpages can be accessed by typing the link into a web browser and reading the information online.

World Health Organisation – Salt Reduction

https://www.who.int/news-room/fact-sheets/detail/salt-reduction

World Action on Salt and Health – The International Low Salt Cookbook

http://www.worldactiononsalt.com/media/action-on-salt/resources/recipe-books/lo-salt-cookbook.pdf

Calories, fat and sodium content in everyday Malaysian favourites

https://www.dietitians.org.my/sites/default/files/webmaster/EduMaterials/What's%20in%20your%20mee%20goreng_calories,%20fat%20&%20sodium%20in%20everyday%20Malaysian%20Favourites.png

DePEC information booklet for high-nitrate vegetable group

DePEC-NUTRITION
(DIETARY NITRATE)

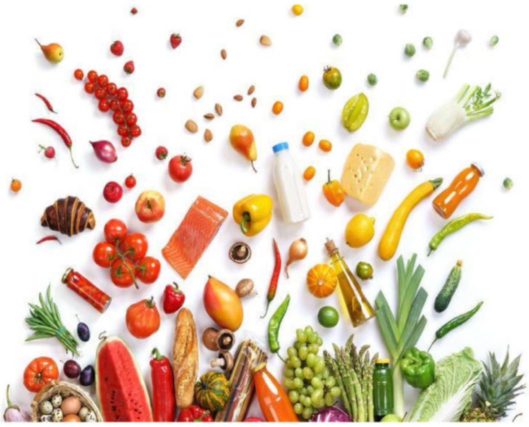

Table of Contents

	Pages
Eating healthily – why is it important?	1
Dietary nitrate – What is it?	2
Health benefits of dietary nitrate	2
Sources of dietary nitrate and recommended intake	2
How to increase your intake of dietary nitrate?	4
Common queries about dietary nitrate	6
Meal suggestions rich in dietary nitrate	10

Eating healthily – why is it important?

Eating well means you are more likely to feel healthier, stay active for longer and protect yourself against illness. It is never too late to start eating healthily, and a healthy diet does not have to be restrictive, boring or expensive.

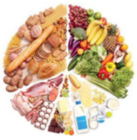

Following a healthy diet has multiple benefits, including: increased energy, improved sleep quality, improved mood and mental wellbeing as well as lowering the risk of chronic health conditions.

In particular, the food we consume is directly related to many of the risk factors for cardiovascular disease – including heart disease, heart failure and stroke - which is one of the main causes of death and disability worldwide. For example, saturated and trans fats increase blood cholesterol which increases your chance of having a heart attack, while polyunsaturated and monounsaturated fats lower the risk of heart attacks. Eating too much salt raises blood pressure, putting extra strain on the cardiovascular system and increasing risk of heart disease, whereas eating more fruit and vegetables can have a preventative effect, as they contain powerful nutrients such as dietary nitrates, which can improve blood pressure and heart health. Furthermore, evidence shows a potential link between the foods we eat and brain processes, such as our thinking or cognitive skills, and as a result by eating more healthily, it can help to reduce the risk of developing dementia.

In this leaflet, we will provide information on one dietary component – dietary nitrate. Research has shown that by **increasing** your intake of nitrate in your diet, you can improve your heart health, especially your blood pressure. This information leaflet will give advice on common sources and recommended intakes, provide practical tips as well as recipes for you to try at home. Take time to read through the material and try to make some changes to your diet based on the advice we have provided.

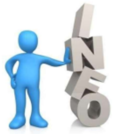

Dietary nitrate – What is it?

Dietary nitrates are compounds found naturally in food and water. Nitrate is an essential plant nutrient that is taken in by all plants and used as their primary nitrogen source. As such, nitrate is a natural part of many fruits and vegetables. In addition, dietary nitrate can also be added to meat products to preserve them for longer period of time. Our bodies convert the nitrate in foods to another small molecule called nitrite, some of which can be further converted into nitric oxide. Nitric oxide is a very important molecule which has a beneficial effect on blood pressure and also on brain function.

Health benefits of dietary nitrate

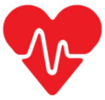

Dietary nitrate has a beneficial role in promoting cardiovascular health, especially by reducing blood pressure and improving blood flow in various organs including the muscle, heart and the brain. As a result, recent research has shown that following a nitrate-rich diet can be an effective strategy to reduce high blood pressure, and therefore can help to protect against the risk of cardiovascular diseases, such as stroke and heart disease.

In addition, by following a nitrate rich diet and therefore regulating your blood pressure, you will also be helping to reduce your risk of developing dementia, in particular vascular dementia. Uncontrolled high blood pressure can cause problems by damaging the blood vessels in your brain, and over time this damage can sometimes affect a person's memory, thinking, or language skills.

Sources of dietary nitrate and recommended intake

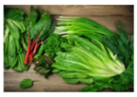

There is a growing body of research highlighting the protective effects of vegetables, particularly green leafy vegetables, such as cabbage, broccoli and spinach, many of which are a very good source of dietary nitrate. Other vegetables that contain a high nitrate amount include beetroot, turnip, pumpkin and we have provided you with a list of these foods later in this document.

To increase nitrate in your diet, we would encourage you to **eat** vegetables that are rich in dietary nitrate **at least three times or more per week**. You should try to eat these foods every day, or on alternate days if you prefer. However, if you

do consume these foods less frequently throughout the week, then you may not receive the desired health benefits that you would be expected to see if you consume them regularly.

If you are able to include these products in your diet as we have suggested, then you are likely to increase your nitrate intake to about **1000-1500mg of dietary nitrate per week** (approximately 200-300mg per day). Just to give you an idea, the usual consumption of dietary nitrate by an individual is around 100-150mg per day. If you will follow our advice, you will be able to double, or even triple, the amount of nitrate that would consume every day in your diet.

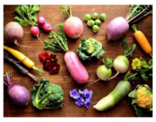

In order to help you with this task, we have first created a table which provides a list of common nitrate rich vegetables typically consumed in Malaysia.

For each vegetable, we have provided the **minimum** amount you are encouraged to eat to achieve this weekly target.

***Please note**: We have included foods that may be consumed by a range of cultures and ethnic groups. Please refer to the foods that are inclusive of your own cultural beliefs.*

**Food item**	**Recommended minimum portion size**
Broccoli *(brokoli)*	150 g (10-12 florets)
Chinese broccoli (*kailan)*	50 g (4 stalks)
Cabbage *(kubis)*; Swamp cabbage (*kangkung*); Chinese cabbage *(kubis cina)*	90 g (1 cup, uncooked)
Celery *(batang saderi)*	50 g (1 medium size stick)
Chinese amaranth *(bayam merah)*	150 g (1 cup)
Eggplant (*terung*)	80 g (1 cup, chopped)
Ginger root *(halia)*	80 g (1/2 cup)
Kale *(kubis kerinting)*	120 g (1 cup, uncooked)
Lettuce *(daun salad)*	60 g (1 cereal bowl)
Pak choi *(sawi)*	60 g (1 cup, shredded)

Pumpkin *(labu)*	160 g (1 cup)
Spinach (bayam)	50 g (1 cereal bowl, uncooked)
Turnip *(sengkuang)*	80 g (1 cup)
Gotu Kola *(pegaga)*	150 g (a bunch, uncooked)
Cauliflower (*kubis bunga*)	150 g (10-12 florets)
Wild pepper leaf (*daun kaduk*)	50 g (1 cereal bowl, uncooked)
Cosmos caudatus (*ulam raja*)	150 g (a bunch, uncooked)
Blackbead (*jering*)	4-5 pieces
Star gooseberry or sweet leaf (*cekur manis*)	200 g (a cup, cooked)
Fern (*pucuk paku*)	200 g (a cup, cooked)

How to increase your intake of dietary nitrate?

In this research study, we hope that you will be able to increase the amount of nitrate consumed in your diet using the information provided in this leaflet. During the study, our researchers will take some measurements to look at nitrate levels in your body. This will help us to see whether an increase in nitrate intake has been achieved.

Tips to help increase your dietary nitrate intake:

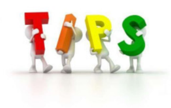

- Vegetables can be fresh green leafy vegetables, as well as canned and frozen varieties
- You can choose both raw or cooked options
- Choose different types of vegetables for lunch and dinner. This will add variety to your meals
- Serve green salads with meals containing lettuce, wild pepper leaf, cosmos caudatusor spinach

- You can cook and prepare these vegetables without affecting the nitrate content. Boil, steam or fry vegetables adding herbs and spices to enhance flavour and taste. Herbs such as coriander, parsley and basil are also rich in dietary nitrates so be generous when cooking with these ingredients. For general health, boiling and steaming are a healthier cooking method rather than frying.
- Eat out smartly. Try to choose dark green vegetable based meals (see some examples on page 4) when eating out. Swap French fries and / or breads for steamed or boiled dark green leafy vegetables or salad when selecting a side / accompanying dish.

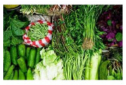

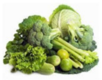

Common queries about dietary nitrate

"Is there a difference between nitrate and nitrite?"

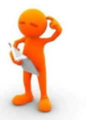

These molecules are different, but are highly correlated by a complex process in the body. Generally, we have both nitrate and nitrite in food. Nitrates are found in generous amounts in green leafy vegetables whereas smaller quantities of nitrites are mostly found in foods such as processed meat products and tinned food. In the body we convert nitrate into nitrite in order to have beneficial effects on our organs, but the body is very good in regulating precisely how much nitrite we need to produce. If the source of nitrite is contaminated water, and in this case nitrite can be harmful if such water sources are consumed. So please avoid drinking or using water for cooking if taken from sources that may not be safe. We would also encourage you to limit the amount of processed foods you consume, therefore opt to increase your dietary nitrate intake by eating foods like green leafy vegetables as we have advised in our leaflet to gain the health benefit.

"Dietary nitrate are present in food and also in water"

The amount contained in water also varies but it is generally lower compared to the amount of nitrate contained in vegetables. Depending on how much nitrate is contained in the water you drink and on how much water you drink in a day, nitrate intake from water may contribute to about 10-15% of the total nitrate intake consumed by an individual. For this study we want to minimise the variability of the sources of nitrate in the water and therefore we advise participants to use the same water for drinking and cooking during the duration of the study.

"Dietary nitrate are used also as preservatives"

Dietary nitrate are used also as preservatives for meat products to increase the shelf life of these products and enhance flavours. There has been speculation around the role of the inorganic nitrate coming from these food sources as risk factor for the onset of cancer. However, these experiments were only done in animals a long time ago and giving high doses and there is no evidence that dietary nitrate intake is associated with cancer risk. Actually, a high vegetable intake is associated with a considerable reduction of the risk for any type of

cancer. Hence, eat your high-nitrate vegetables and it will reduce your risk of cancer in the long term.

"The cooking method affects how much nitrate is left in the food"

This is possible, but the effect on the nitrate content of the food that is ultimately eaten after cooking will be minimal. Hence, do not worry too much about the cooking method as long as you have included as much high-nitrate vegetables as you can in your diet. However, try to choose healthier cooking methods such as steaming, boiling or grilling.

"How much nitrate can I have in my diet until it becomes unsafe?"

As for any nutrient we have set upper limits of consumption of nitrate in humans and we say that we should not consume more than 2 grams of nitrate per day. However, this is an incredibly high amount and your consumption during the study if you increase your vegetable intake will be around 8-10 fold lower (~0.2-0.3 grams per day). Just to put things into perspective, you will need to eat about 4kilograms of cabbage to reach a consumption more than 2grams per day!

"Is nitrate absorbed better by my body if the food is in a liquid form?"

This is not true and you will absorb nitrate in the same way if your food is in a solid or liquid form. So eat your vegetables as you like the most as long as you include them in your diet.

"Does nitrate affect or is affected by other nutrients in the body?"

It is unlikely that nitrate will affect the properties of other nutrients in the diet. However, we know for example that citrus fruits or berries may help the beneficial effects of nitrate on the body. So if you eat your vegetables, continue your healthy choices by adding a piece of fruit to the meal and the benefits of nitrate will be increased. Finally, we only know that smoking affects how much nitrate is absorbed in the body and therefore smokers will receive less benefits from nitrate in the vegetables even if the eat their portion of vegetables.

"Does washing my teeth with normal toothpaste affect the conversion of nitrate in the body?"

No, there is no evidence that brushing your teeth will affect the beneficial effects that you will receive from the nitrate in the vegetables. However, mouthwash use will affect the conversion of nitrate in the body, and therefore we advise you that during the study you will avoid or limit the use of mouthwash to not affect the results of the study. Ask the research team if you want more information on this.

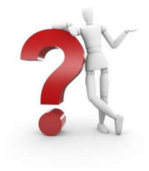

Meal suggestions rich in dietary nitrate

Here are some examples of nitrate rich meals that you can prepare at home:

Kerabu Pegaga

Key nitrate ingredient: Gotu Kola (*Pegaga*)

Bahan bahan:

1 ikat pegaga - dihiris
1-2 biji limau nipis
2 batang serai – hiris

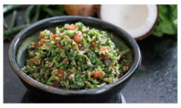

Bahan tumbuk:

1 inci halia - hiris
4 ulas bawang putih - hiris
1 sudu kecil lada hitam
Segenggam ikan bilis

Cara Penyediaan:

1. *Goreng semua bahan tumbuk tanpa minyak sehingga garing. Setelah hampir garing, baru dimasukkan serai yang dihiris. Goreng lagi sehingga bahan lain garing dan agak-agak boleh ditumbuk.*
2. *Tumbuk kasar atau kisar menggunakan* blender *kering sehingga hancur tapi jangan terlalu hancur.*
3. *Hiris pegaga dan masukkan ke dalam mangkuk. Tuang bahan kisar kasar tadi keatasnya. Perahkan air limau nipis secukupnya. Gaul rata dan sedia untuk dihidangkan.*

Stir Fried Prawn and Fern Salad

Key nitrate ingredient: Fern (*Pucuk paku*)

Bahan-bahan:

3 ikat pucuk paku
10 ekor udang saiz besar

1 camca besar sos tiram
1 camca sos ikan secukup rasa
1/3 cawan air

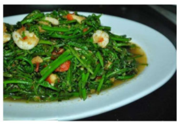

Bahan tumbuk:

1 biji bawang besar
2 ulas bawang putih
1 biji cili hijau
1 biji cili merah
1/2 kiub pati ikan bilis

Cara Penyediaan:

1. *Pucuk paku diasingkan dari tangkainya, cuci dan toskan. Udang dikupas kulit, cuci bersih dan toskan. Panaskan 3 camca besar minyak dalam kuali, tumis bahan-bahan tumbuk hingga naik bau, guna api kecil dan kacau selalu.*
2. *Masukkan sos tiram, kacau dan tambah udang. Masak hingga udang bertukar warna. Tambah air, dan biar sekejap. Masukkan pucuk paku dan gaul rata. Masak hingga pucuk paku lembut, tapi bukan sampai lembik. Tambah sos ikan secukup rasa, kacau dan masak sekejap.*

Sayur Kubis Goreng (Stir Fried Cabbage)

Key nitrate ingredient: Cabbage (*Kubis*)

Bahan-bahan:

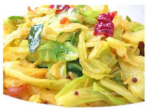

1/2 biji kubis (sederhana besar) – potong memanjang
1/2 biji bawang besar - hiris
2 ulas bawang putih - ketuk
2 biji lada cili api - ketuk
5 ekor udang kering - tumbuk
Secubit garam

Cara Penyediaan:

1. *Tumis bawang besar, bawang putih dan udang kering hingga garing kekuningan.*
2. *Masukkan kubis.*
3. *Gaul rata.*
4. *Masukkan sedikit air.*
5. *Perasakan dengan garam.*
6. *Gaul sebati dan biarkan mereneh sekejap.*
7. *Bila kuah hampir mengering, tutup api.*
8. *Angkat dan hidangkan.*

Sayur Bayam Tumis Air (Spinach Soup)

Key nitrate ingredient: Spinach

Bahan-bahan:

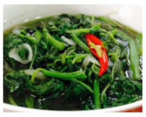

1 ikat sayur bayam - di siang, asingkan daunnya dan tangkainya.
6 ekor udang
4 ulas bawang merah - di hiris
2 ulas bawang putih - di hiris
1 inci halia - di hiris
1 batang cili merah - di hiris
Garam secukup rasa
Air secukupnya

Cara Penyediaan:

1. *Panaskan sedikit minyak.*
2. *Tumiskan bahan hiris kecuali cili merah dan masak hingga naik bau.*
3. *Tambahkan udang dan air.*
4. *Biarkan kuah merenih.*
5. *Perasakan dengan garam.*
6. *Masukkan batang bayam dahulu.*
7. *Bila dah lembut masukkan sayur bayam dan cili merah.*
8. *Tutup api dan kacau rata.*
9. *Sedia di hidangkan.*

Kailan Ikan Masin (Chinese Broccoli in Salted Fish)

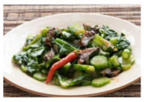

Key nitrate ingredient: Chinese broccoli

Bahan-Bahan:

1 ikat sayur kailan
3 ulas bawang putih, kupas kulit dan ketuk (garlic)
4 biji cili padi, ketuk (chili)
1 keping kecil isiikan masin, potong kecil (salted fish – soak to reduce sodium content)
1 sudu besar sos tiram kurang garam (light oyster sauce)
2 sudu besarminyak (cooking oil)

Cara Penyediaan:

1. *Basuh dan rendam sayur kailan di dalam air sehingga daunnya tampak segar. Potong daun sepanjang 1 inci, batang sayur pula dihiris nipis.*
2. *Panaskan minyak, tumis bawang putih sehingga naik bau dan masukkan cili padi beserta isi ikan masin. Kacau seketika.*
3. *Masukkan pula bahagian batang kalian dan sos tiram, kemudian diikuti bahagian daun.*
4. *Tuang sedikit air sebagai kuah.*
5. *Angkat dan hidang.*

Kerabu Ulam Raja (*Cosmos caudatus*)

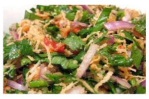

Key nitrate ingredient: *Cosmos caudatus*

Bahan-bahan:

1 ikat ulam raja
½ cawan kelapa parut (grated coconut)
2 sudu makan perahan limau kasturi (lime juice)
1 labu bawang besar (Dihiris) (onion)
3 biji cili merah (red chili)
4 biji cili api (bird chili)
4 biji bawang merah
1 sudu makan udang kering (dried shrimp)
(Cili merah, cili api, bawang merah dan udang kering dikisar bersama) – chili, onion, dried shrimp to be blend together)

Cara Penyediaan:

1. *Basuhkan ulam raja dan potong halus.*
2. *Goreng kelapa parut sehingga kekuningan sahaja.*
3. *Masukkan semua bahan ke dalam mangkuk dan gaul rata.*
4. *Sedia dihidangkan.*

Broccoli with Lemon Butter Sauce

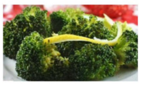

<u>Key nitrate ingredient:</u> Broccoli

Bahan-Bahan:

¼ *cawan mentega* (butter)
¼ *cawan air*
1 biji perahan jus lemon
¼ *sudu teh* cayenne
Lada hitam secukup rasa (black pepper)
400gm brokoli

Cara Penyediaan:

1. *Masukkan mentega, air, jus lemon, lada* cayenne, *lada hitam ke dalam kuali.*
2. *Biarkan mereneh atas api yang sederhana.*
3. *Masukkan brokoli, kacau dan tutup.*
4. *Masak antara 10 – 15 minit (api sederhana).*
5. *Biarkan ia masak hingga lembut namun masih mengekalkan warna hijaunya.*
6. *Hidangkan panas-panas.*

Masak Lemak Pucuk Manis dengan Keledek

<u>Key nitrate ingredient</u>: Sweet leaf

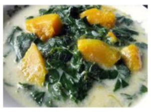

Bahan-bahan:

2 ikat daun pucuk manis
3 biji keledek
9 biji cili api
5 biji bawang kecil
2 ulas bawang putih
Ikan bilis
1 inci kunyit hidup
Santan
Sedikit garam

Cara Penyediaan:

1. *Tumbuk cili api, bawang merah, bawang putih, ikan bilis, garam dan kunyit hidup sampai lumat, ketepikan.*
2. *Tumis bahan yang telah di tumbuk tadi.*
3. *Setelah naik bau masukkan keledek dan santan.*
4. *Bila keledek sudah empuk masukkan daun pucuk manis dan garam.*
5. *Biarkan seketika. Bila sudah masak angkat dan boleh dihidangkan.*

Further Reading

We have provided some further reading below to supplement the information described in this leaflet. These webpages can be accessed by typing the link into a web browser and reading the information online.

Kings College London - Eat your greens: understanding the cardiovascular benefits of dietary nitrate

https://www.kcl.ac.uk/news/spotlight-article?id=75a75805-0c5c-4fcf-98a1-4f591dcbd8db

BBC Online - The truth about the nitrates in your food

http://www.bbc.com/future/story/20190311-what-are-nitrates-in-food-side-effects

DePEC information booklet for combined high-nitrate vegetable and reduce-salt group

DePEC – NUTRITION
(SALT AND DIETARY NITRATE)

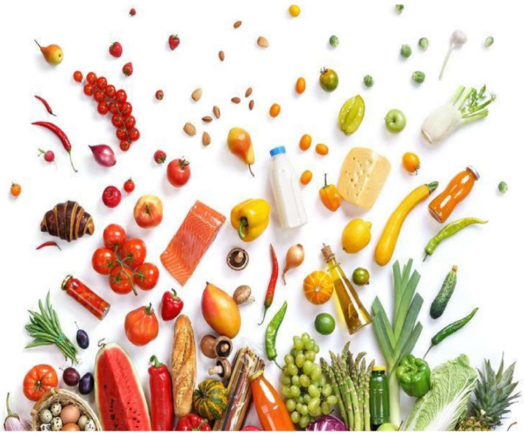

Table of Contents

	Pages
Eating healthily – why is it important?	1
Salt – What is it?	2
Salt and Health	2
Recommended Intake	3
Common Sources of Salt in Malaysia	4
How to be more salt aware?	7
Reading Food Labels	8
Salt content of common condiments used in Malaysia	10
Food Swaps	11
Common misperceptions about salt reduction	12
Salt Measuring Spoon	13
How to use the salt measuring spoon?	13
Meal suggestions low in salt	15
Dietary nitrate – What is it?	21
Health benefits of dietary nitrate	21
Sources of dietary nitrate and recommended intake	21
How to increase your intake of dietary nitrate?	23
Common queries about dietary nitrate	25
Meal suggestions rich in dietary nitrate	29

Eating healthily – why is it important?

Eating well means you are more likely to feel healthier, stay active for longer and protect yourself against illness. It is never too late to start eating healthily, and a healthy diet does not have to be restrictive, boring or expensive.

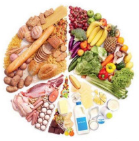

Following a healthy diet has multiple benefits, including: increased energy, improved sleep quality, improved mood and mental wellbeing as well as lowering the risk of chronic health conditions.

In particular, the food we consume is directly related to many of the risk factors for cardiovascular disease – including heart disease, heart failure and stroke - which is one of the main causes of death and disability worldwide. For example, saturated and trans fats increase blood cholesterol which increases your chance of having a heart attack, while polyunsaturated and monounsaturated fats lower the risk of heart attacks. Eating too much salt raises blood pressure, putting extra strain on the cardiovascular system and increasing risk of heart disease, whereas eating more fruit and vegetables can have a preventative effect, as they contain powerful nutrients such as dietary nitrates, which can improve blood pressure and heart health. Furthermore, evidence shows a potential link between the foods we eat and brain processes, such as our thinking or cognitive skills, and as a result by eating more healthily, it can help to reduce the risk of developing dementia.

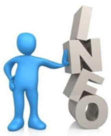

In this leaflet, we will provide information on two dietary components – salt and dietary nitrate. Research has shown that by **reducing** the amount of salt you eat, and **increasing** your intake of nitrate in your diet, you can improve your heart health, especially your blood pressure. This information leaflet will give advice on common sources and recommended intakes, provide practical tips as well as recipes for you to try at home. Take time to read through the material and try to make some changes to your diet based on the advice we have provided.

Salt – What is it?

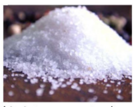

Salt is the everyday term we use for a chemical compound called sodium chloride.

Although it is not a requirement, some manufacturers may still quote the sodium content of foods on their labels. It is important to know that **each gram of sodium translates to 2.5 grams** of salt, and to take this into account when looking at your everyday salt consumption.

Many everyday foods are not obviously salty, but they can contain high amounts of 'hidden salt'. While we may add salt to our food during cooking or just before we eat it, a large amount of the salt we eat is already added to the food we buy. Most of this comes from salt added to foods like processed meat products, snacks (such as crackers and biscuits), soups and sauces, including soy sauce.

Salt and Health

Reducing salt intake has been identified as one of the most cost-effective measures countries can take to improve the health of their population. While sodium is essential for muscle and cell function, eating too much salt raises blood pressure and increases the risk of developing heart disease and having a stroke.

High blood pressure puts added force against the walls of your arteries (the blood vessels that carry blood around the body). Over time, this extra pressure can damage the arteries, which makes them more likely to become narrowed and hardened by fatty deposits. When this happens it can increase the risk of having angina or a heart attack.

Research has also shown that having high blood pressure can also increase your risk of developing dementia, in particular vascular dementia. Uncontrolled high blood pressure can cause problems by damaging the blood vessels in your brain. Over time, this raises the risk of a blood vessel becoming blocked or bursting. If a blood vessel cannot carry energy and oxygen to a part of the brain properly, some cells in the brain may be

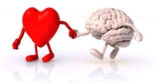

damaged. This damage can sometimes affect a person's memory, thinking, or language skills.

Recommended Intake

Average salt intake among Malaysians is around 7.15 grams per day, exceeding the World Health Organisation (WHO) recommendations.

WHO recommends a reduction **to less than 2 grams of sodium per day**, which is equivalent to **5 grams of salt per day** - this is less than one teaspoon of salt per day.

Common Sources of Salt in Malaysia

The most common foods that are high in salt in Malaysia, with low salt alternatives, have been listed below. Increase the number of lower salt foods in your diet and limit high salt options within the same food group. ***Please note:*** *We have included foods that may be consumed by a range of cultures and ethnic groups. Please refer to the foods that are inclusive of your own cultural beliefs.*

Common foods HIGH in salt	LOW salt options	Salt Reduction GOALS
**Condiments and Spices**		
Kicap cair **(Light soy sauce)**<br>***Kicap pekat*** **(Dark soy sauce)**<br>***Sambal belacan*** **(Shrimp paste)**<br>***Sos tiram*** **(Oyster sauce)**<br>***Sos cili/ tomato*** **(Chilli/ tomato ketchup)**<br>***Budu*** **(Anchovies paste)**<br>**Mayonnaise / Salad dressing**<br>**Monosodium Glutamate**<br>***Tempoyak*** **(fermented durian)**<br>***Taucu*** **(fermented soybean paste)**<br>**Instant seasoning powder**	All natural condiments, herbs and spices (such as cloves, cinnamon, anise seeds, cumin seeds, turmeric, *asam gelugor*, cardamom, dried chilli, thyme, basil, ginger, curry powder)<br>Reduced salt soy sauce<br>Tomato puree<br>Lower sodium substitutes such as potassium salt	Replace the high salt condiments with more low salt options to add flavour to meals. E.g. you could choose reduced salt soy sauce instead of the standard varieties.<br><br>Reduce the number of times you use the high salt condiments when cooking e.g. if you use every day, try using them only 3-4 times a week.<br><br>Measure - use measuring spoons when cooking e.g. if usually add 4 tablespoons of soy sauce to a meal, try gradually reducing the number of tablespoons you add over a few weeks. Your taste buds will adapt to the lower salt taste
**Rice and Carbohydrate Sources**		
Nasi goreng **(Fried rice)**<br>***Nasi lemak*** **(Rice cooked in coconut milk, garnished with fried anchovies, peanut, egg, cucumber)**	Rice or noodles, plain, boiled<br>Bread, white or wholemeal<br>Biscuit, soda/plain or low salt, whole-wheat crackers	Reduce the consumption of salt while preparing or cooking these foods at home. You can do this by using measuring spoons to measure the amount of salt you add to foods/meals when cooking e.g. if you normally

Common foods HIGH in salt	LOW salt options	Salt Reduction GOALS
**Meat and Meat Products**		
Sup daging lembu **(Mutton soup)**<br>***Ayam goreng berempah*** **(Spicy fried chicken)**<br>**Chicken, fried, fast food franchise**<br>**Chicken curry, canned**<br>**Chicken broth cubes (pati ayam)**<br>**Beef burger with cheese**<br>**Beef *rendang*, canned**<br>**Mutton curry, canned**<br>**Meat extract (Bovril / Marmite)**	Choose fresh, unprocessed chicken, beef or mutton when preparing meat based meals at home	Reduce the consumption of salt while preparing and/or cooking these foods at home.<br><br>Replace salt with low salt condiments, herbs and spices to add flavour to vegetables when cooking.
**Fish**		
**High salt content:**<br>***Ikan masak sambal*** **(Fried fish with sauce)**<br>***Bebola kek*** **(Fish ball)**<br>**Fish, dried, salted**<br>**Fish Sauce**<br>**Anchovy, dried, without head and entrails**<br>**Sardine, canned**<br>**Shrimp, fermented (*Cencaluk*)**<br>**Shrimp paste (*Belacan*)**<br><br>**Moderate salt content:**<br>**Fish Crackers, fried**<br>**Prawn, salted, dried**<br>**Prawn crackers**<br>**Prawn paste (*Hay-Ko*)**	Choose fresh, unprocessed fish and prawns when preparing fish based meals at home	Reduce the consumption of salt while preparing and/or cooking these foods at home. Be aware of fish sauces as they can have a high salt content.<br><br>Replace salt with low salt condiments, herbs and spices to add flavour to vegetables when cooking.

How to be more salt aware?

In this research study, we hope that you will be able to reduce the amount of salt you eat using the information provided in this leaflet. During the study, our researchers will take some measurements to look at sodium levels in your body. This will help us to see whether a reduction in salt intake has been achieved.

Here are a few practical tips to help you reduce the level of salt in your diet:

- Reduce the amount of salt added in cooking and the addition of other flavour enhancers such as monosodium glutamate (MSG), sauces (such as soy sauce, oyster sauce, fish sauce, tomato sauce) and flavouring cubes. Avoid using table salt or adding sauces while enjoying the food.

- Enhance the flavour of food using natural herbs and condiments such as garlic, onion, curry spices, white pepper, lemon grass, vinegar and lemon.

Here are some examples of herbs and spices which can enhance the flavour of different foods:

- Basil: Use with beef, chicken and most vegetables
- Curry: Use with beef, chicken, fish, green beans, carrots and in marinades
- Ginger: Use with beef, chicken, green beans, cauliflower and egg plant
- Thyme: Use with beef, chicken, fish, green beans, beets and carrots

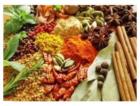

- Learn to enjoy natural flavour of foods without salt

- Reduce intake of salty foods such as salted fish, salted eggs, salted vegetables, high-sodium snacks (such as potato crisps and chips) and processed foods (such as sausages, chicken nuggets, meatballs and burgers / burger patty)

- Choose fresh fruits and vegetables instead of preserved and processed types. Reduce/avoid using sour plum powder, mayonnaise and salad dressing when enjoying fruits and vegetables

❖ Soak preserved foods such as dried anchovies in water to reduce sodium content

❖ Choose low sodium breakfast cereal e.g. porridge, wheat biscuits or bran flakes

❖ Drain the water from the can before consuming canned product because it is high in salt, which is used to preserve the food

❖ Eat out smartly. Eating out while controlling your sodium intake is a challenge because the sodium content of a food served is always unknown and often is higher than expectation. Therefore, it is advisable to try not eating out too often. Limit fast food consumption and request for low salt and less sauces dishes or no MSG added meals when eating out

[illegible]

- Avoid adding extra salt or sauces to foods such as tomato, chilli and soy sauce
- Choose more freshly prepared options rather than processed foods like sausages, bacon and hot dog as these are likely to be high in salt
- Choose steamed vegetables as a side dish rather than French fries, for example

Reading Food Labels

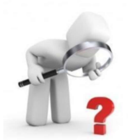

Have a look at the sodium content of a food in the Nutrition Information Panel (NIP). This is a table on the food label which lists all the major nutrients contained in the product. Compare the sodium content with other available brands of the same product and choose the ones with the lower sodium content. Try to choose brands with "low" or "lower" salt claims on the label, if available.

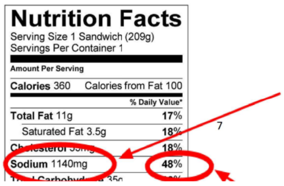

This is an example of a Nutrition Information Panel.

You will see that in 209g (1 serving) of this food product, there is 1140mg (1.14g) of sodium, or 2.85g of salt (1.14g sodium x 2.5).

This product is HIGH in salt, containing more than half of the [illegible]

Also, look out for the following ingredients on the food label which contain sodium:

- Monosodium glutamate (MSG)
- Sodium nitrate
- Sodium benzoate

Salt content of common condiments used in Malaysia

Below are some examples of condiments used in Malaysia with their estimated salt content per tablespoon. These have been labelled use in moderation (to consume every so often), or use sparingly (to consume in small amounts, less often).

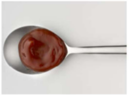

Tomato Ketchup
0.3g salt in one tablespoon serving.
One table spoon contributes towards 6% of your recommended daily salt intake of 5g.
Use in moderation

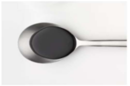

Soy Sauce
2.75g salt in one tablespoon serving.
One tablespoon contributes towards 55% of your recommended daily salt intake of 5g.
Use sparingly

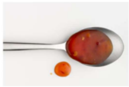

Chilli / Hot sauce
0.6g salt in one tablespoon serving.
One tablespoon contributes towards 12% of your recommended daily salt intake of 5g.
Use in moderation

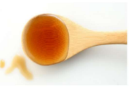

Fish sauce
2.65g salt in one tablespoon serving.
One tablespoon contributes towards 53% of your recommended daily salt intake of 5g.
Use sparingly

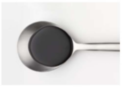

Oyster sauce
1.0g salt in one tablespoon serving
One tablespoon contributes towards 20% of your recommended daily salt intake of 5g.
Use sparingly

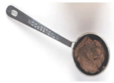

Anchovy paste
2.7g salt in one tablespoon serving
One tablespoon contributes towards 54% of your recommended daily salt intake of 5g.
Use sparingly

Food Swaps

Here are some simple food swaps you can try in order to reduce your salt intake:

HIGH SALT	LOW SALT
Fried/Curry Chicken	Roasted/grilled Chicken
Salted Nuts	Unsalted Nuts
Cream Cracker	Low-Salt Wholewheat Cracker
Cooked/fried vegetables	Fresh/raw vegetables (*ulam-ulaman*)
Fried fish	Steamed/grilled fish

Common misperceptions about salt reduction

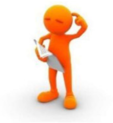

"On a hot and humid day when you sweat, you need more salt in the diet"

There is little salt lost through sweat so there is no need for extra salt even on a hot and humid day, although it is important to drink a lot of water

"Sea salt is not 'better' than manufactured salt simply because it is 'natural'"

Regardless of the source of salt, it is the sodium in salt that causes bad health outcomes

"Salt added during cooking is the main source of salt intake"

In many countries, about 80% of salt in the diet actually comes from processed foods or food eaten outside the home, for example in restaurants

"Food requires salt to have appealing flavour"

It takes some time for a person's taste buds to adjust, but once they get used to less salt, one is more likely to enjoy food and notice a broader range of flavours

"Food has no flavour without salt"

Whilst food will initially taste different without salt, taste buds soon become accustomed to less salt and you are more likely to enjoy food with less salt, and more flavour

"Foods high in salt taste salty"

Some foods that are high in salt don't taste very salty because sometimes they are mixed with other things like sugars that mask the taste. It is important to read food labels to find out sodium levels

"Only old people need to worry about how much salt they eat"

Eating too much salt can raise blood pressure at any age

"Reducing salt could be bad for my health"

It's very difficult to eat too little salt since there are so many everyday foods containing salt

Salt Measuring Spoon

We have provided you with a salt measuring spoon to help you measure the amount of salt that is recommended for daily consumption by WHO (5g per day).

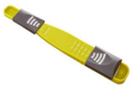

As mentioned, approximately 80% of salt consumed comes from **processed foods.** It is important to take this into consideration in relation to the total 5g daily intake, as this will include all **<u>salt added during cooking in the home, salt already added during processing or added in preparation of meals when eating out in a restaurant or takeaway</u>.** Therefore, it is important to read food labels or ask staff in restaurants to find out about salt content of foods.

How to use the salt measuring spoon?

1. Transfer the salt you have at home into a container that is easily accessible, e.g. a container that has a wide opening
2. Adjust the measuring spoon to the 5g setting. This is the **total amount** of salt one person can have in one day. Alternatively, by selecting the 1g setting on the spoon, you can use **5 x 1g portions per day**. From the salt container, scoop salt using the spoon and level off.
3. Add the salt to meals when cooking to your desired taste, keeping within the 5g daily allowance.

When cooking for more than one person within the household, simply multiply the daily limit by the number of people, e.g. if cooking meals for a family of 3, the total daily allowance of salt would be 15g.

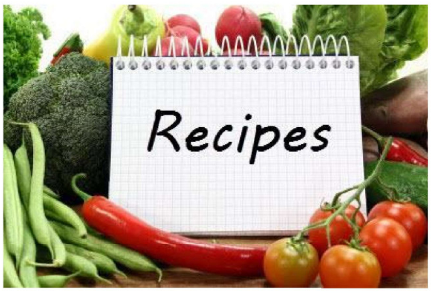

Meal suggestions low in salt

Here are some examples of low salt meals that you can prepare at home:

Vegetable *Mee Goreng* (Fried Noodles) (Serves 3-4)

Ingredients:

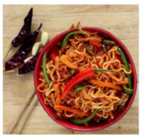

3 tablespoons cooking oil e.g palm oil
2 cloves garlic finely minced
3 pieces dried bean curd cut into small pieces
1 potato boiled, peeled, and sliced into wedges
1 big tomato cut into wedges
A handful fresh bean sprouts
1 lb fresh yellow noodles rinsed and drained
2 red chillies for garnishing, chopped
2 shallots for garnishing, chopped
1 lime cut into wedges

Sauce:
2 tablespoon *reduced salt* soy sauce
3 tablespoon tomato puree

Instructions:

1. Heat up a wok and pour in the cooking oil. Add the garlic and stir fry until aromatic then add the bean curd and potato and tomato wedges. Do a few quick stirs and then add the yellow noodles and the sauce and continue stirring until everything is well combined.
2. Add in the bean sprouts and quick stir for another 1 minute. Turn off the heat, dish out, garnish with the red chili and shallots. Squeeze some lime juice over the noodles before eating.

Spiced Rice with Chicken (Serves 4-6)

Ingredients:

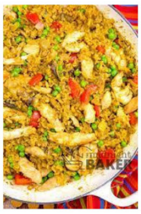

900g chicken
1 clove garlic
5cm piece of ginger
2 red chillies
1 tbsp poppy seeds
10 cashew nuts, plain, unsalted
10 almonds
2 tbsp ghee
5 cloves
5cm cinnamon stick
1 cup shallots, sliced
1 tsp curry powder
1/2 cup yoghurt, plain
1-2 cups coconut milk
400g rice

Instructions:

1. Cut chicken into pieces. Grind together garlic, ginger, chillies, poppy seeds, cashew nuts and almonds.
2. Heat ghee and fry cloves, cinnamon stick and shallots. Add chicken pieces, ground ingredients and curry powder. Stir to mix and cook covered for 10 minutes. Add yoghurt and simmer till chicken is tender and mixture is thick.
3. Add coconut milk to the rice and cook. When rice has absorbed all the coconut milk make a well in the centre and place the chicken mixture in the centre. Cover and allow the rice to finish cooking over a very low heat. Serve.

Hot and Sour Malay Fish Curry (Serves 4)

Ingredients:

2 tablespoon of cooking oil e.g. palm oil
1 cm galangal root, bruised
3-4 slices *asam gelugur* or 1 ½ heaped tablespoons tamarind pulp soaked in 4 tablespoons water, mashed and strained to obtain juice
625ml water
6 thick fresh fish fillets (about 750g)
4 sprigs laksa leaves (*daun kesum*), minced
3 tablespoons thick coconut milk
1 teaspoon sugar
1 tablespoon pineapple achar

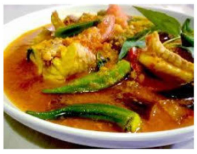

Spice paste:
2 candlenuts, roughly chopped
10 chillies, cut into lengths
10 shallots, peeled and halved
4 cloves of garlic, peeled and halved
½ teaspoon turmeric
250 ml water

Instructions:

1. Grind the spice paste ingredients to a paste in a mortar or blender, adding a little oil if necessary to keep the blades turning.
2. Heat the oil in a pot over a medium heat and stir fry the spice paste and galangal for 5 minutes until fragrant. Add the *asam gelugur* or tamarind juice and ½ cup (125ml) water, and cook for 5 minutes.
3. Add the rest of the water and bring to a boil. Then add the fish, laksa leaves, coconut milk and sugar. Simmer, uncovered, for another 5-10 minutes until the oil separates from the coconut milk. Serve with freshly cooked white rice.

Chicken *Rendang* (Serves 4)

Ingredients:

220g boneless chicken breast (raw), cut into bite size pieces

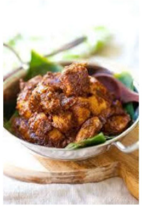

Blend the following ingredients until fine:
3 shallots, chopped
4 cloves garlic
3 whole fresh red chilies
1 inch ginger
2 sticks lemongrass
1 inch galangal
1 inch ground turmeric
15 cm x 1 pc turmeric leaves, slice finely
5 cm x 3 pcs kaffir lime leaves, slice finely
2 tablespoons cooking oil e.g palm oil
1⁄2 cup water

Instructions:

1. Heat oil in a heavy bottom pan or a pot. Sauté the blended spices for a minute or until fragrant.
2. Put the chicken and water in the pan or pot, and stir over medium heat and bring to a boil.
3. Reduce heat to a simmer and cook until the gravy thickens. Add turmeric leaves and kaffir lime leaves. Continue to cook until the meat becomes tender and the gravy reduced to a desired thickness.
4. Serve hot in individualized portion during meal time.

Lentil and Vegetable Curry (Serves 4)

Ingredients:

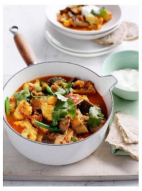

Olive oil spray or 1 tablespoon cooking oil e.g palm oil
1 large onion, thinly sliced
200g mushrooms, halved
2 garlic cloves, crushed
400g eggplant, cut into 2.5 cm dice
1-2 tablespoons korma curry paste
600g cauliflower, trimmed, cut into small florets
1 x 400g can no added salt tomatoes
1 cup reduced salt vegetable or chicken stock or water
1 x 400g can brown lentils, rinsed and drained
150g green beans, topped, halved
1/2 cup reduced fat evaporated milk
1/2 cup chopped flat leaf parsley or coriander
4 small wholemeal pita breads and 4 tablespoons reduced fat natural yoghurt, to serve

Instructions:

1. Heat a large, deep non-stick frying pan or saucepan over medium heat. Spray with oil. Add onion and cook, stirring often, for 3-4 minutes or until the onion is soft and light golden. Remove to a plate. Spray pan lightly again with oil. Add the mushrooms and garlic, cook, stirring often for 2-3 minutes or until mushrooms are lightly browned. Remove to the plate with the onions.
2. Spray pan lightly again with oil. Add eggplant and cook, stirring often for 3-4 minutes or until just softened. Add 2 teaspoons of water to create steam if the eggplant is sticking. Return the onions, mushrooms and garlic to the pan. Add curry paste, stir to coat, cook for 1 minute.
3. Stir in cauliflower, tomatoes, and stock or water. Bring to boil. Reduce the heat to low and simmer, covered for 10-15 minutes. Add lentils and beans and simmer, uncovered for a further 5-10 minutes or until beans are just cooked through. Stir through the evaporated milk and parsley or coriander.

4. Serve with pita bread and yoghurt if desired.

Tip: You can replace eggplant with sweet potato, pumpkin or regular potatoes.

Further Reading

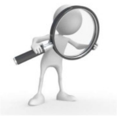

We have provided some further reading below to supplement the information described in this leaflet. These webpages can be accessed by typing the link into a web browser and reading the information online.

World Health Organisation – Salt Reduction

https://www.who.int/news-room/fact-sheets/detail/salt-reduction

World Action on Salt and Health – The International Low Salt Cookbook

http://www.worldactiononsalt.com/media/action-on-salt/resources/recipe-books/lo-salt-cookbook.pdf

Calories, fat and sodium content in everyday Malaysian favourites

https://www.dietitians.org.my/sites/default/files/webmaster/EduMaterials/What's%20in%20your%20mee%20goreng_calories,%20fat%20&%20sodium%20in%20everyday%20Malaysian%20Favourites.png

Dietary nitrate – What is it?

Dietary nitrates are compounds found naturally in food and water. Nitrate is an essential plant nutrient that is taken in by all plants and used as their primary nitrogen source. As such, nitrate is a natural part of many fruits and vegetables. In addition, dietary nitrate can also be added to meat products to preserve them for longer period of time. Our bodies convert the nitrate in foods to another small molecule called nitrite, some of which can be further converted into nitric oxide. Nitric oxide is a very important molecule which has a beneficial effect on blood pressure and also on brain function.

Health benefits of dietary nitrate

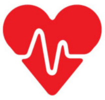

Dietary nitrate has a beneficial role in promoting cardiovascular health, especially by reducing blood pressure and improving blood flow in various organs including the muscle, heart and the brain. As a result, recent research has shown that following a nitrate-rich diet can be an effective strategy to reduce high blood pressure, and therefore can help to protect against the risk of cardiovascular diseases, such as stroke and heart disease.

In addition, by following a nitrate rich diet and therefore regulating your blood pressure, you will also be helping to reduce your risk of developing dementia, in particular vascular dementia. Uncontrolled high blood pressure can cause problems by damaging the blood vessels in your brain, and over time this damage can sometimes affect a person's memory, thinking, or language skills.

Sources of dietary nitrate and recommended intake

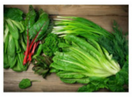

There is a growing body of research highlighting the protective effects of vegetables, particularly green leafy vegetables, such as cabbage, broccoli and spinach, many of which are a very good source of dietary nitrate. Other vegetables that contain a high nitrate amount include beetroot, turnip, pumpkin and we have provided you with a list of these foods later in this document.

To increase nitrate in your diet, we would encourage you to **eat** vegetables that are rich in dietary nitrate **at least three times or more per week**. You should try to eat these foods every day, or on alternate days if you prefer. However, if you

do consume these foods less frequently throughout the week, then you may not receive the desired health benefits that you would be expected to see if you consume them regularly.

If you are able to include these products in your diet as we have suggested, then you are likely to increase your nitrate intake to about **1000-1500mg of dietary nitrate per week** (approximately 200-300mg per day). Just to give you an idea, the usual consumption of dietary nitrate by an individual is around 100-150mg per day. If you will follow our advice, you will be able to double, or even triple, the amount of nitrate that would consume every day in your diet.

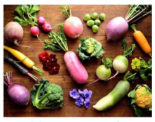

In order to help you with this task, we have first created a table which provides a list of common nitrate rich vegetables typically consumed in Malaysia.

For each vegetable, we have provided the **minimum** amount you are encouraged to eat to achieve this weekly target.

Please note*: We have included foods that may be consumed by a range of cultures and ethnic groups. Please refer to the foods that are inclusive of your own cultural beliefs.*

Food item	Recommended minimum portion size
Broccoli *(brokoli)*	150 g (10-12 florets)
Chinese broccoli (*kailan)*	50 g (4 stalks)
Cabbage *(kubis)*; Swamp cabbage (*kangkung*); Chinese cabbage *(kubis cina)*	90 g (1 cup, uncooked)
Celery *(batang saderi)*	50 g (1 medium size stick)
Chinese amaranth *(bayam merah)*	150 g (1 cup)
Eggplant (*terung*)	80 g (1 cup, chopped)
Ginger root *(halia)*	80 g (1/2 cup)
Kale *(kubis kerinting)*	120 g (1 cup, uncooked)
Lettuce *(daun salad)*	60 g (1 cereal bowl)
Pak choi *(sawi)*	60 g (1 cup, shredded)

Pumpkin *(labu)*	160 g (1 cup)
Spinach (bayam)	50 g (1 cereal bowl, uncooked)
Turnip (*sengkuang*)	80 g (1 cup)
Gotu Kola *(pegaga)*	150 g (a bunch, uncooked)
Cauliflower (*kubis bunga*)	150 g (10-12 florets)
Wild pepper leaf (*daun kaduk*)	50 g (1 cereal bowl, uncooked)
Cosmos caudatus (*ulam raja*)	150 g (a bunch, uncooked)
Blackbead (*jering*)	4-5 pieces
Star gooseberry or sweet leaf (*cekur manis*)	200 g (a cup, cooked)
Fern (*pucuk paku*)	200 g (a cup, cooked)

How to increase your intake of dietary nitrate?

In this research study, we hope that you will be able to increase the amount of nitrate consumed in your diet using the information provided in this leaflet. During the study, our researchers will take some measurements to look at nitrate levels in your body. This will help us to see whether an increase in nitrate intake has been achieved.

Tips to help increase your dietary nitrate intake:

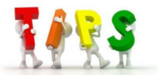

- Vegetables can be fresh green leafy vegetables, as well as canned and frozen varieties
- You can choose both raw or cooked options
- Choose different types of vegetables for lunch and dinner. This will add variety to your meals
- Serve green salads with meals containing lettuce, wild pepper leaf, cosmos caudatusor spinach

- ❖ You can cook and prepare these vegetables without affecting the nitrate content. Boil, steam or fry vegetables adding herbs and spices to enhance flavour and taste. Herbs such as coriander, parsley and basil are also rich in dietary nitrates so be generous when cooking with these ingredients. For general health, boiling and steaming are a healthier cooking method rather than frying.

- ❖ Eat out smartly. Try to choose dark green vegetable based meals (see some examples on page 4) when eating out. Swap French fries and / or breads for steamed or boiled dark green leafy vegetables or salad when selecting a side / accompanying dish.

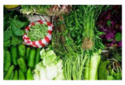

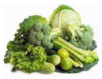

Common queries about dietary nitrate

"Is there a difference between nitrate and nitrite?"

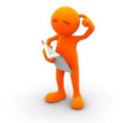

These molecules are different, but are highly correlated by a complex process in the body. Generally, we have both nitrate and nitrite in food. Nitrates are found in generous amounts in green leafy vegetables whereas smaller quantities of nitrites are mostly found in foods such as processed meat products and tinned food. In the body we convert nitrate into nitrite in order to have beneficial effects on our organs, but the body is very good in regulating precisely how much nitrite we need to produce. If the source of nitrite is contaminated water, and in this case nitrite can be harmful if such water sources are consumed. So please avoid drinking or using water for cooking if taken from sources that may not be safe. We would also encourage you to limit the amount of processed foods you consume, therefore opt to increase your dietary nitrate intake by eating foods like green leafy vegetables as we have advised in our leaflet to gain the health benefit.

"Dietary nitrate are present in food and also in water"

The amount contained in water also varies but it is generally lower compared to the amount of nitrate contained in vegetables. Depending on how much nitrate is contained in the water you drink and on how much water you drink in a day, nitrate intake from water may contribute to about 10-15% of the total nitrate intake consumed by an individual. For this study we want to minimise the variability of the sources of nitrate in the water and therefore we advise participants to use the same water for drinking and cooking during the duration of the study.

"Dietary nitrate are used also as preservatives"

Dietary nitrate are used also as preservatives for meat products to increase the shelf life of these products and enhance flavours. There has been speculation around the role of the inorganic nitrate coming from these food sources as risk factor for the onset of cancer. However, these experiments were only done in animals a long time ago and giving high doses and there is no evidence that dietary nitrate intake is associated with cancer risk. Actually, a high vegetable intake is associated with a considerable reduction of the risk for any type of

cancer. Hence, eat your high-nitrate vegetables and it will reduce your risk of cancer in the long term.

"The cooking method affects how much nitrate is left in the food"

This is possible, but the effect on the nitrate content of the food that is ultimately eaten after cooking will be minimal. Hence, do not worry too much about the cooking method as long as you have included as much high-nitrate vegetables as you can in your diet. However, try to choose healthier cooking methods such as steaming, boiling or grilling.

"How much nitrate can I have in my diet until it becomes unsafe?"

As for any nutrient we have set upper limits of consumption of nitrate in humans and we say that we should not consume more than 2 grams of nitrate per day. However, this is an incredibly high amount and your consumption during the study if you increase your vegetable intake will be around 8-10 fold lower (~0.2-0.3 grams per day). Just to put things into perspective, you will need to eat about 4kilograms of cabbage to reach a consumption more than 2grams per day!

"Is nitrate absorbed better by my body if the food is in a liquid form?"

This is not true and you will absorb nitrate in the same way if your food is in a solid or liquid form. So eat your vegetables as you like the most as long as you include them in your diet.

"Does nitrate affect or is affected by other nutrients in the body?"

It is unlikely that nitrate will affect the properties of other nutrients in the diet. However, we know for example that citrus fruits or berries may help the beneficial effects of nitrate on the body. So if you eat your vegetables, continue your healthy choices by adding a piece of fruit to the meal and the benefits of nitrate will be increased. Finally, we only know that smoking affects how much nitrate is absorbed in the body and therefore smokers will receive less benefits from nitrate in the vegetables even if the eat their portion of vegetables.

"Does washing my teeth with normal toothpaste affect the conversion of nitrate in the body?"

No, there is no evidence that brushing your teeth will affect the beneficial effects that you will receive from the nitrate in the vegetables. However, mouthwash use will affect the conversion of nitrate in the body, and therefore we advise you that during the study you will avoid or limit the use of mouthwash to not affect the results of the study. Ask the research team if you want more information on this.

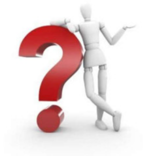

Meal suggestions rich in dietary nitrate

Here are some examples of nitrate rich meals that you can prepare at home:

Kerabu Pegaga

Key nitrate ingredient: Gotu Kola (*Pegaga*)

Bahan bahan:

1 ikat pegaga - dihiris
1-2 biji limau nipis
2 batang serai – hiris

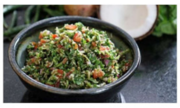

Bahan tumbuk:

1 inci halia - hiris
4 ulas bawang putih - hiris
1 sudu kecil lada hitam
Segenggam ikan bilis

Cara Penyediaan:

1. *Goreng semua bahan tumbuk tanpa minyak sehingga garing. Setelah hampir garing, baru dimasukkan serai yang dihiris. Goreng lagi sehingga bahan lain garing dan agak-agak boleh ditumbuk.*
2. *Tumbuk kasar atau kisar menggunakan* blender *kering sehingga hancur tapi jangan terlalu hancur.*
3. *Hiris pegaga dan masukkan ke dalam mangkuk. Tuang bahan kisar kasar tadi keatasnya. Perahkan air limau nipis secukupnya. Gaul rata dan sedia untuk dihidangkan.*

Stir Fried Prawn and Fern Salad

Key nitrate ingredient: Fern (*Pucuk paku*)

Bahan-bahan:

3 ikat pucuk paku
10 ekor udang saiz besar

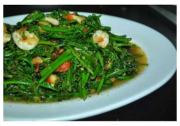

1 camca besar sos tiram
1 camca sos ikan secukup rasa
1/3 cawan air

Bahan tumbuk:

1 biji bawang besar
2 ulas bawang putih
1 biji cili hijau
1 biji cili merah
1/2 kiub pati ikan bilis

Cara Penyediaan:

1. *Pucuk paku diasingkan dari tangkainya, cuci dan toskan. Udang dikupas kulit, cuci bersih dan toskan. Panaskan 3 camca besar minyak dalam kuali, tumis bahan-bahan tumbuk hingga naik bau, guna api kecil dan kacau selalu.*
2. *Masukkan sos tiram, kacau dan tambah udang. Masak hingga udang bertukar warna. Tambah air, dan biar sekejap. Masukkan pucuk paku dan gaul rata. Masak hingga pucuk paku lembut, tapi bukan sampai lembik. Tambah sos ikan secukup rasa, kacau dan masak sekejap.*

Sayur Kubis Goreng (Stir Fried Cabbage)

Key nitrate ingredient: Cabbage (*Kubis*)

Bahan-bahan:

1/2 biji kubis (sederhana besar) – potong memanjang
1/2 biji bawang besar - hiris
2 ulas bawang putih - ketuk
2 biji lada cili api - ketuk
5 ekor udang kering - tumbuk
Secubit garam

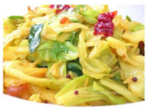

Cara Penyediaan:

1. *Tumis bawang besar, bawang putih dan udang kering hingga garing kekuningan.*
2. *Masukkan kubis.*
3. *Gaul rata.*
4. *Masukkan sedikit air.*
5. *Perasakan dengan garam.*
6. *Gaul sebati dan biarkan mereneh sekejap.*
7. *Bila kuah hampir mengering, tutup api.*
8. *Angkat dan hidangkan.*

Sayur Bayam Tumis Air (Spinach Soup)

Key nitrate ingredient: Spinach

Bahan-bahan:

1 ikat sayur bayam - di siang, asingkan daunnya dan tangkainya.
6 ekor udang
4 ulas bawang merah - di hiris
2 ulas bawang putih - di hiris
1 inci halia - di hiris
1 batang cili merah - di hiris
Garam secukup rasa
Air secukupnya

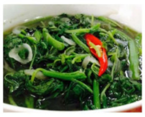

Cara Penyediaan:

1. *Panaskan sedikit minyak.*
2. *Tumiskan bahan hiris kecuali cili merah dan masak hingga naik bau.*
3. *Tambahkan udang dan air.*
4. *Biarkan kuah merenih.*
5. *Perasakan dengan garam.*
6. *Masukkan batang bayam dahulu.*
7. *Bila dah lembut masukkan sayur bayam dan cili merah.*
8. *Tutup api dan kacau rata.*
9. *Sedia di hidangkan.*

Kailan Ikan Masin (Chinese Broccoli in Salted Fish)

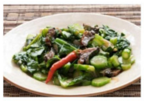

Key nitrate ingredient: Chinese broccoli

Bahan-Bahan:

1 ikat sayur kailan
3 ulas bawang putih, kupas kulit dan ketuk (garlic)
4 biji cili padi, ketuk (chili)
1 keping kecil isiikan masin, potong kecil (salted fish – soak to reduce sodium content)
1 sudu besar sos tiram kurang garam (light oyster sauce)
2 sudu besarminyak (cooking oil)

Cara Penyediaan:

1. *Basuh dan rendam sayur kailan di dalam air sehingga daunnya tampak segar. Potong daun sepanjang 1 inci, batang sayur pula dihiris nipis.*
2. *Panaskan minyak, tumis bawang putih sehingga naik bau dan masukkan cili padi beserta isi ikan masin. Kacau seketika.*
3. *Masukkan pula bahagian batang kalian dan sos tiram, kemudian diikuti bahagian daun.*
4. *Tuang sedikit air sebagai kuah.*
5. *Angkat dan hidang.*

Kerabu Ulam Raja (*Cosmos caudatus*)

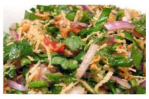

Key nitrate ingredient: *Cosmos caudatus*

Bahan-bahan:

1 ikat ulam raja
½ cawan kelapa parut (grated coconut)
2 sudu makan perahan limau kasturi (lime juice)
1 labu bawang besar (Dihiris) (onion)
3 biji cili merah (red chili)
4 biji cili api (bird chili)
4 biji bawang merah
1 sudu makan udang kering (dried shrimp)
(Cili merah, cili api, bawang merah dan udang kering dikisar bersama) – chili, onion, dried shrimp to be blend together)

Cara Penyediaan:

1. *Basuhkan ulam raja dan potong halus.*
2. *Goreng kelapa parut sehingga kekuningan sahaja.*
3. *Masukkan semua bahan ke dalam mangkuk dan gaul rata.*
4. *Sedia dihidangkan.*

Broccoli with Lemon Butter Sauce

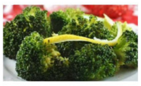

Key nitrate ingredient: Broccoli

Bahan-Bahan:

¼ cawan mentega (butter)
¼ cawan air
1 biji perahan jus lemon
¼ sudu teh cayenne
Lada hitam secukup rasa (black pepper)
400gm brokoli

Cara Penyediaan:

1. *Masukkan mentega, air, jus lemon, lada* cayenne, *lada hitam ke dalam kuali.*
2. *Biarkan mereneh atas api yang sederhana.*
3. *Masukkan brokoli, kacau dan tutup.*
4. *Masak antara 10 – 15 minit (api sederhana).*
5. *Biarkan ia masak hingga lembut namun masih mengekalkan warna hijaunya.*
6. *Hidangkan panas-panas.*

Masak Lemak Pucuk Manis dengan Keledek

Key nitrate ingredient: Sweet leaf

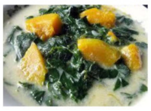

Bahan-bahan:

2 ikat daun pucuk manis
3 biji keledek
9 biji cili api
5 biji bawang kecil
2 ulas bawang putih
Ikan bilis
1 inci kunyit hidup
Santan
Sedikit garam

Cara Penyediaan:

1. *Tumbuk cili api, bawang merah, bawang putih, ikan bilis, garam dan kunyit hidup sampai lumat, ketepikan.*
2. *Tumis bahan yang telah di tumbuk tadi.*
3. *Setelah naik bau masukkan keledek dan santan.*
4. *Bila keledek sudah empuk masukkan daun pucuk manis dan garam.*
5. *Biarkan seketika. Bila sudah masak angkat dan boleh dihidangkan.*

Further Reading

We have provided some further reading below to supplement the information described in this leaflet. These webpages can be accessed by typing the link into a web browser and reading the information online.

Kings College London - Eat your greens: understanding the cardiovascular benefits of dietary nitrate

https://www.kcl.ac.uk/news/spotlight-article?id=75a75805-0c5c-4fcf-98a1-4f591dcbd8db

BBC Online - The truth about the nitrates in your food

http://www.bbc.com/future/story/20190311-what-are-nitrates-in-food-side-effects

Text message reminders for DePEC salt intervention group

Month	End of week 1	End of week 3
Month 1	Hi [participant’s name]. Have you read the information leaflet on salt we gave you at clinic? This provides useful advice on salt and health, recommended salt intake and practical tips on how to eat less salt. Have a look, think about the sources of salt in your own diet and make a start to eat less salt from today.	Hi [participant’s name]. Most people consume too much salt. On average, people consume around twice the recommended daily maximum limit (5g). Eating less than 5 grams per day for adults (just under 1 teaspoon) helps to reduce blood pressure and risk of cardiovascular disease, stroke and coronary heart attack. Have a look at our leaflet for further information on the health benefits of eating less salt.
Month 2	Hi [participant’s name]. Try to reduce the amount of salt added in cooking at home. Use the measuring spoon provided to measure out the recommended daily salt limit of 5g. From this amount, you can add this salt to any meals that you prepare that day. When cooking for more than one person within the household, simply multiply the daily allowance (5g) by the number of people.	Hi [participant’s name]. Most of the salt we eat every day is "hidden". In many countries, about 80% of salt in the diet comes from processed foods like condiments, sauces, bread, biscuits and breakfast cereals, and prepared ready meals or takeaways. Try to reduce your intake of these foods. Have a look at our salt information leaflet for practical tips and low salt food options.
Month 3	Hi [participant’s name]. Salt or sodium? Some food labels may only state the sodium content. Don't confuse salt and sodium figures. To convert sodium to salt, you need to multiply the sodium amount by 2.5. For example, 1g of sodium per 100g is 2.5 grams of salt per 100g. Have a read through our salt information leaflet for further advice.	Hi [participant’s name]. Remember to check the salt content on food labels. The Nutrition Information Panel (NIP) lists all the major nutrients contained in the product. Compare the sodium content with other available brands of the same product and choose the ones with the lower salt content. Try to choose brands with "low" or "lower" salt claims on the label, if available.
Month 4	Hi [participant’s name]. Some people think that food has no flavour without salt. Whilst this may be true at first, your taste buds will soon become accustomed to less salt and you are more likely to enjoy food with less salt, and more flavour. Don’t give up!	Hi [participant’s name]. You can prepare flavoursome meals at home without adding salt. Why not try adding things like fresh, frozen or dried herbs, onions, garlic, shallots, chillies, ginger, cinnamon, lemon juice, pepper or vinegar to meals and recipes. Check out our salt information leaflet for practical tips and recipes you can try.

Month 5	Hi [participant’s name]. Remember to eat out smartly. Eating out while controlling your salt intake is a challenge because the salt content of food served is always unknown and often is higher than we think. Therefore, it is advisable to try not eating out too often. Tip: ask for your sauces and dressings to be served ‘on the side’ so that you can choose how much to use. Remember to read our salt information leaflet for more tips!	Hi [participant’s name]. Remember to check the salt content on food labels when out shopping. At first, you will find that it will take you a little longer to find the brands and foods that contain the lowest amount of salt when you are shopping. Write a list of low-salt brands and keep it with you. Once you have discovered the lowest salt choices, then shopping will be just as quick as it used to - but much more salt-free!
Month 6	Hi [participant’s name]. Have you tried any of the recipes in our salt information leaflet? These have been developed as a low salt option, using herbs and spices to add flavour rather than salt or by reducing the amount of condiments, like soy sauce, added during cooking. You can try this approach with other recipes you traditionally prepare at home. Have a look at our information leaflet for further tips and advice.	Hi [participant’s name]. You have now been taking part in this research study for 6 months and have reached the end of the intervention period – well done! The changes you have made to reduce salt in your diet will have big health benefits. This is a lifestyle change and we would encourage you to continue to reduce to the amount of salt in your diet – don’t give up!

Text message reminders for the DePEC nitrate intervention group

**Month**	**End of week 1**	**End of week 3**
Month 1	Hi [participant’s name]. Have you read the information leaflet on dietary nitrate we gave you at clinic? This provides useful advice on the health benefits of a nitrate-rich diet, recommended intakes and practical tips on how to eat more dietary nitrate. Have a look, think about the sources of nitrate in your own diet and make a start to increase your intake from today.	Hi [participant’s name]. Research has shown that following a nitrate-rich diet can be an effective strategy to reduce high blood pressure, and can help to protect against the risk of cardiovascular diseases in the future. Have a look at our information leaflet for further advice of the health benefits of following a diet rich in nitrates.
Month 2	Hi [participant’s name]. Vegetables, particularly dark, green leafy vegetables, such as cabbage, broccoli and spinach, are an excellent source of dietary nitrate. Other vegetables such as beetroot, turnip, celery and pumpkin are also very good sources of nitrate. Have a look at our information leaflet for more examples and recommended minimum portion sizes.	Hi [participant’s name]. We would encourage you to consume nitrate-rich vegetables at least three times or more per week. You should try to eat these foods every day, or on alternate days if you prefer. If you do consume these foods less frequently throughout the week, then you may not receive the desired health benefits that you would be expected to see if you consume them regularly. Have a think about the number of times you would eat these vegetables per week, and try to increase your intake from today.
Month 3	Hi [participant’s name]. You can cook and prepare nitrate rich vegetables without affecting the nitrate content. Boil, steam or fry vegetables, adding herbs and spices to enhance flavour and taste. Herbs such as coriander, parsley and basil are also rich in dietary nitrates so be generous when cooking with these ingredients. For general health, boiling and steaming are a healthier cooking method rather than frying. Remember to read our information leaflet for further tips and advice.	Hi [participant’s name]. When trying to follow a more nitrate-rich diet, you can choose vegetables that are fresh green leafy vegetables, as well as frozen varieties. You can also choose both raw and cooked options. Choose different types of vegetables for lunch and dinner. This will add variety to your meals. Check out our information leaflet for ideas of nitrate rich vegetables that you can try.

Month 4	Hi [participant's name]. Does nitrate affect, or is affected by, other nutrients in the body? We know that citrus fruits or berries may help the beneficial effects of nitrate on the body. So if you eat your vegetables, continue your healthy choices by adding a piece of fruit to the meal and the benefits of nitrate will be increased. Smoking can affect how much nitrate is absorbed in the body and therefore smokers will receive less benefits from nitrate in the vegetables even if the eat their portion of vegetables.	Hi[participant's name]. Remember in order to gain the most health benefit from following a nitrate-rich diet, try to eat dark, green leafy vegetables at least 3 times a week. You should try to eat these foods every day, or on alternate days if you prefer. Vegetables like spinach, cabbage, broccoli, celery and kale are excellent sources. Remember to read our information leaflet on dietary nitrate for more ideas and tips.
Month 5	Hi [participant's name]. Have you tried any of the recipes in our dietary nitrate information leaflet? These incorporate key nitrate rich vegetables. At home, you can prepare nitrate rich meals by including plenty of dark green leafy vegetables and herbs when cooking. Have a look at our information leaflet for further tips and advice.	Hi [participant's name]. Remember to eat out smartly. Try to choose dark green vegetable-based meals when eating out. Swap French fries and / or breads for steamed or boiled dark green leafy vegetables or green leafy salad when selecting a side / accompanying dish.
Month 6	Hi [participant's name]. Everyone can benefit from the health and nutritional benefits of eating more green leafy vegetables. Encourage your family members to do the same. Have a look at our information leaflet for some recipe ideas that you can prepare for the whole family.	Hi [participant's name]. You have now been taking part in this research study for 6 months and have reached the end of the intervention period – well done! The changes you have made to increase nitrate in your diet will have big health benefits. This is a lifestyle change and we would encourage you to continue to follow a nitrate-rich diet – don't give up!

The salt measuring spoon given to the participants from low salt consumption and combined high-nitrate vegetable plus low salt consumptions)

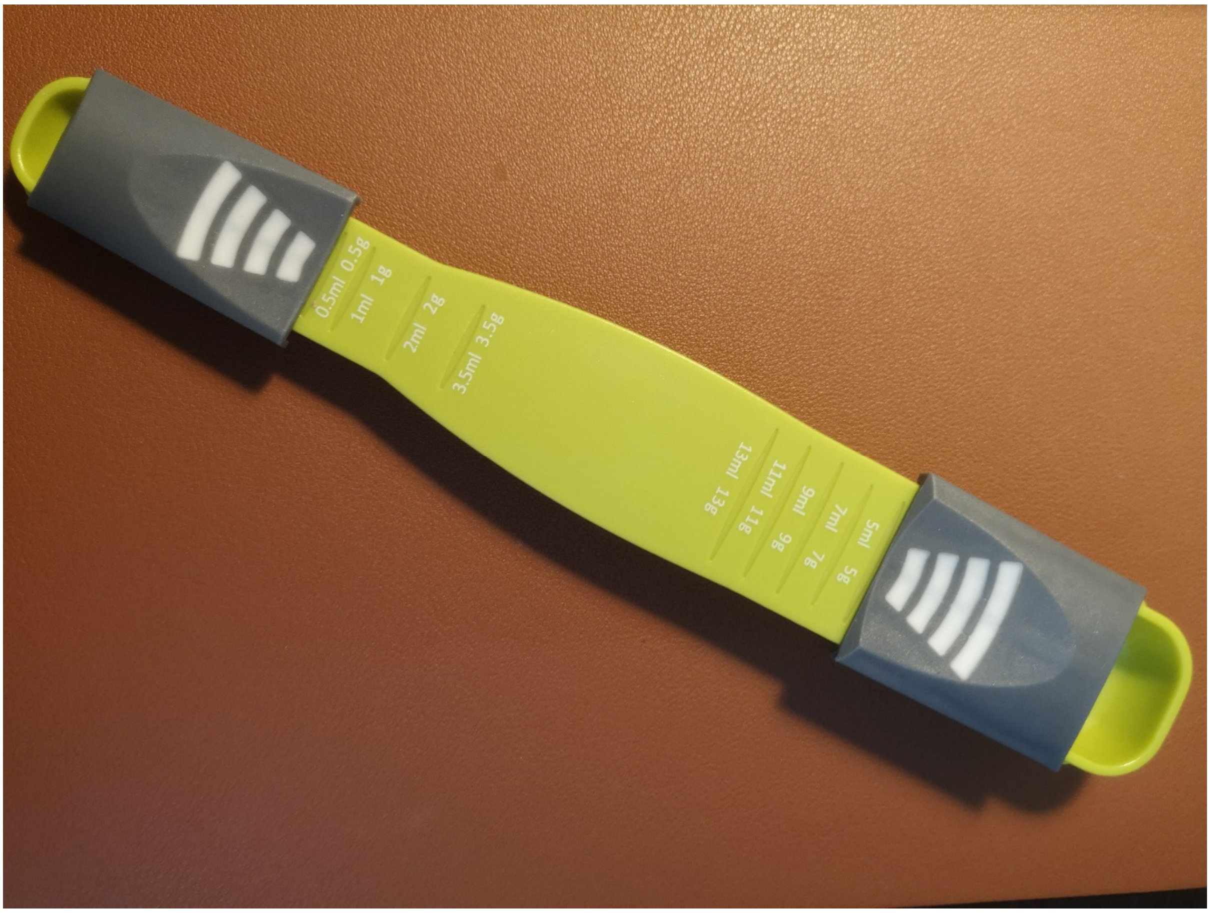

DePEC-Nutrition study questionnaire

ADHERENCE QUESTIONNAIRE

Screening ID:

Data Collector's Name:

Date & Time:

Session: Interim 1 / Interim 2

Hello, my name is xxx and I am a Data Collector from SEACO, Monash University. I am conducting this phone interview as now is not a suitable time for us to visit you face to face. We would like to ask you a few questions to better understand your experiences of the DePEC Nutrition study. This will take around 10-15 minutes. Can I proceed?

If No, set an appointment for another day.

If Yes,

Thank you for agreeing to participate. So there are no right or wrong answers to any of our questions, we are interested in your own opinions and experiences. All responses will be kept confidential. Are there any questions about what I have just explained?

Let's begin...

Can I audio record this interview? This is to ease my record and follow up, the audio record will be kept confidentially. Yes □ No □

1. Have you experienced any side effects during the past 3 months (1st interim)/ 5 months (2nd interim)? Yes □ No □

1a. If so, what are they?

____

____

1b. If yes, do you think they are related to the study? Yes □ No □

Justification:

____

____

____

2. On a scale between 0 (no compliance) to 10 (high compliance), could you provide an indicative score on the level of compliance to the allocated nutritional intervention(s)? Score:

3. Could you please identify what have been the main challenges and barriers you had to face that limited the ability to follow your allocated nutritional intervention?

4. Did you experience any challenges with accessibility of food items during the COVID-19 pandemic? Yes □ No □

If so, can you describe which items in particular?

____

____

____

5. Do you think the COVID-19 pandemic affected your ability to comply with the intervention?

Yes □ No □

If so, please explain why?

____

____

____

6. I would like to ask you about the materials we have provided. On a scale between 0 (clear) to 10 (not clear), could you provide an indicative score on the clarity of the specific material provided to you to follow the allocated nutritional intervention(s)? Score:

6a. Could you please briefly explain the motivations of your score?

7. Could you please indicate the main dietary changes you have made during the study in relation to your allocated nutritional intervention?

8. You will have received text message reminders twice a week with specific dietary reinforcement advice for your allocated intervention. Have you read these text messages? Yes □ No □

8a. On a scale between 0 (no helpful at all) to 10 (very helpful), could you provide an indicative score on how helpful was the information received with the text messages in helping you to comply with the allocated nutritional intervention(s)? Score:

8b. Could you please briefly explain the motivations of your score?

9. Finally, do you have any additional comments you would like to add that we may not have discussed already? Additional comments:

We have now finished the telephone interview.

DePEC Study Evaluation – Part 1 (Researcher's Copy)

Combined Intervention Group

Date:

Time:

Respondent ID:

[Turn on audio recorder]

[Introduction]

Hi. Mr/ Mdm__________. I am ______, from SEACO.

Thank you for agreeing to participate in this telephone interview. Today is the first session of the interview.

During the interview, you will have to refer to the Participant Information Sheet (PIS) and questionnaire which we posted to you earlier. Do you have the materials with you now?

[If response = No, WhatsApp PIS and part 1 questionnaire to participant]

[If response = **YES**, continue the interview]

May I turn on the audio recorder?

This is a recording of part 1 telephone interview for participant 00x from combined intervention group.

As mentioned, over the past 10 months, you have been taking part in a research study to ***reduce salt and increase dietary nitrate***.

[Questionnaire]

[Participation]

1. Think back to when you first became involved with this research study. What were the main reasons for your participation in this study?

   ____

[Evaluation of the DePEC intervention materials]

2. Information Leaflet
   a. Did you find the written information leaflet that we provided useful? (Please indicate your score from 1 being not useful to 5 being very useful.)

Not useful	*Slightly useful*	*Moderately useful*	*Useful*	*Very useful*
1	2	3	4	5

b. Was the information easy to understand?
(Please indicate your score from 1 being very difficult to 5 being very easy.)

Very difficult	*Difficult*	*No strong opinion*	*Easy*	*Very easy*
1	2	3	4	5

c. Did you find the information relevant to you?
(Please indicate your score from 1 being not relevant to 5 being very relevant.)

Not relevant	*Slightly relevant*	*Moderately relevant*	*Relevant*	*Very relevant*
1	2	3	4	5

d. Did you find the information culturally appropriate?
(Please indicate your score from 1 being not appropriate to 5 being very appropriate.)

Not appropriate	*Slightly appropriate*	*Moderately appropriate*	*Appropriate*	*Very appropriate*
1	2	3	4	5

e. Did you access the additional material suggested in the further reading section (last page of the booklet)?

Yes ☐ No ☐

f. Did you try and prepare any of the recipes in the booklet?

Yes ☐ No ☐

If no, why not? ____

3. Additional Resources

a. Did you use the salt measuring spoon we provided (PIS Appendix, page 4)?

Yes ☐ No ☐

b. If yes, did you find the spoon easy to use?
(Please indicate your score from 1 being very difficult to 5 being very easy.)

Very difficult	*Difficult*	*No strong opinion*	*Easy*	*Very easy*
1	2	3	4	5

4. Text and Video Messages

a. How useful were the text message reminders?
(Please indicate your score from 1 being not useful to 5 being very useful.)

Not useful	*Slightly useful*	*Moderately useful*	*Useful*	*Very useful*
1	2	3	4	5

b. Do you think the frequency of messages was appropriate? (i.e. two messages per week)
(Please indicate your score from 1 being not appropriate to 5 being very appropriate.)

Not appropriate	*Slightly appropriate*	*Moderately appropriate*	*Appropriate*	*Very appropriate*
1	2	3	4	5

c. During the study you were visited / contacted by a researcher who shared a video of key advice in relation to reduce salt and increase dietary nitrate. Did you find this useful? (Please indicate your score from 1 being not useful to 5 being very useful.)

Not useful	*Slightly useful*	*Moderately useful*	*Useful*	*Very useful*
1	2	3	4	5

Counselling Sessions at KK

a. Did you find the counselling session useful at the beginning of the study? (Please indicate your score from 1 being not useful to 5 being very useful.)

Not useful	*Slightly useful*	*Moderately useful*	*Useful*	*Very useful*
1	2	3	4	5

b. Can you please explain briefly some reasons for your answer?

____
____

5. Overall, do you have any suggestions for improving the advice we give to future participants? Please comment:

____
____

Food tolerance and preparation

6. Based on your experience, please tell us how acceptable it was to change your diet to eat less salt? (Please indicate your score from 1 being very unacceptable to 5 being very acceptable.)

Very unacceptable	*Unacceptable*	*No strong opinion*	*Acceptable*	*Very acceptable*
1	2	3	4	5

7. How would you rate the taste of food with using less salt? (Please indicate your score from 1 being very poor to 5 being very good.)

Very poor	*Poor*	*No strong opinion*	*Good*	*Very good*
1	2	3	4	5

8. Do you often eating out or consume meals outside of the home?

Yes □ No □

Can you please briefly explain some reasons for your answer?

____

9. Do you plan to continue to follow our dietary recommendations (i.e. eat less salt and more high-nitrate food)?

Yes □ No □

If no, why not? ____

10. Would you recommend participation in a similar study to your family and friends?
Yes □ No □
If no, why not? ____

11. If we are to undertake this study again using computerised technology to provide dietary education instead of a traditional printed leaflet e.g. on tablet, ipad, would you be interested?
Yes □ No □
If no, why not? ____

Study design

12. Was the number of visits appropriate?
[1] Appropriate [2] Too many [3] Too few
If 2 or 3, please explain why: ____
13. Was the location of the visits at the clinic convenient?
Yes □ No □
If no, where would you prefer? ____

14. Was the duration of the visits acceptable?
[1] Appropriate [2] Too long [3] Too short
If 2 or 3 please explain why: ____

15. During the study you completed a series of measurements. The following question asks you to rate your experience of these measurements.
Please tick the response that most applies to you in relation to your experience of:

	Very difficult or burdensome	***Difficult or burdensome***	***No strong opinion***	***Easy or straightforward***	***Very easy or straightforward***
Providing the blood samples					
Having blood pressure taken					
Having weight and height measurements taken					
Providing a saliva sample					

Close of interview and appointment for second interview session

Is there anything else you would like to add that we haven't already discussed? Is there anything else you would like to ask?
[Check with the participant if there are any particular statements that they do not wish to be transcribed].
Thank you so much for taking the time to do this interview. When are you free for us to continue the second part of the interview?
